# Author Correction: Intracellular localization of nanoparticle dimers by chirality reversal

**DOI:** 10.1038/s41467-018-06060-0

**Published:** 2018-08-27

**Authors:** Maozhong Sun, Liguang Xu, Joong Hwan Bahng, Hua Kuang, Silas Alben, Nicholas A. Kotov, Chuanlai Xu

**Affiliations:** 1 State Key Laboratory of Food Science and Technology, Jiangnan University, Wuxi, 214122 China; 2 International Joint Research Laboratory for Biointerface and Biodetection, Jiangnan University, Wuxi, 214122 China; 3 Chemical Engineering Department, University of Michigan, Ann Arbor, MI 48109 USA; 4 Department of Biomedical Engineering, University of Michigan, Ann Arbor, MI 48109 USA; 5 Department of Mathematics, University of Michigan, Ann Arbor, MI 48109 USA; 6 Department of Material Sciences and Engineering, University of Michigan, Ann Arbor, MI 48109 USA; 7 Michigan Center for Integrative Research in Critical Care, Ann Arbor, MI 48109 USA; 8 Biointerfaces Institute, University of Michigan, Ann Arbor, MI 48109 USA

Correction to: *Nature Communications* 10.1038/s41467-017-01337-2; published online 29 Nov 2017

The original version of this Article contained an error in the spelling of the author Joong Hwan Bahng, which was incorrectly given as Joong Hwan Banhg. This has been corrected in both the PDF and HTML versions of the Article.

