# Supplementary material for: Intracellular localization of nanoparticle dimers by chirality reversal
Source: Nat Commun. 2017 Nov 29;8:1847. doi: 10.1038/s41467-017-01337-2 (PMC5707389; doi:10.1038/s41467-017-01337-2)
Supplement: Supplementary file 1 — Supplementary Information [file 41467_2017_1337_MOESM1_ESM.pdf]

Supplementary Methods:

Self-assembly of NP/NR dimers: 20 nm Au NPs were synthesized according to the classical citrate reduction method[1,2] and then re-dispersed in 0.01 M phosphate buffer (PB) at pH 7.4. After that, phosphorothioate-modified DNA1 and DNA2 were added into two NP dispersions with a DNA-to-NP ratio of 5:1, respectively. Subsequently, 0.05 M NaCl was mixed and then incubated for 12 h at ambient temperature of 25 ºC, and then the excess DNA was removed though centrifugation at 8500 rpm for 15 min. Finally, DNA1-NP and DNA2-NP were mixed for 12 h incubation at ambient temperature to form the NP dimers.

Samples of gold nanorods (NRs) with aspect ratios of 3 and 2.5 were synthesized by the gold seed mediated growth methods and re-dispersed in 5 mM cetyltrimethylammonium bromide (CTAB) buffer with a final concentration of 5 nM. Subsequently, phosphorothioate-modified DNA1 and DNA2 were added to two NR dispersions with DNA-to-NR ratios of 50:1, respectively. The excess DNA was removed though centrifugation, and then, the above NR-DNA1 and NR-DNA2 solutions were mixed. After incubation for 12 h at ambient temperature (25 ºC), NR dimers were obtained. The detailed sequences of the oligonucleotide were (the underlined nucleotides are phosphorothioate-modified):

DNA 1: 5′ -<u>CAA</u>TAGCCCTTGGATAAAAAAA<u>AAA</u>-SH-3′

DNA 2: 5′ -<u>ATC</u>CAAGGGCTATTGAAAAAAA<u>AAA</u>-SH-3′

Quantitation of DNA linker number per NPs: The concentration of thiol-modified DNA attached to each NP was estimated as follows:

1) FAM-modified complementary DNA3 (7μL, 10μM) was added to 5 μL of the purified 20 nm Au NP-DNA1 probes solution. The sequence of 5(6)-carboxyfluorescein (FAM)-modified complementary DNA3 was 5'-ATCCAAGGGCTATTGAAAAAAAAAA-FAM-3'. The solution was heated to 70 °C for 5 min, and then cooled to room temperature. We could measure the fluorescence signal of the hybridization of the above solution after two hours.
2) Determine a standard curve that relates the concentration of a fluorescently labeled DNA and fluorescence intensity using the fluorescence maximums of different concentration of DNA-FAM.
3) The concentration of FAM modified complementary DNA could be calculated by measuring the fluorescence maximums based on the standard calibration curve. So, the average DNA1 on each NP could be determined.
4) As similar to the above method for detecting the concentration of DNA2 modified on the surface of Au NPs, using the standard fluorescence standard calibration curve, we also obtained the DNA2 concentration onto the surface of Au NPs. And the sequence of FAM-DNA4 was 5'-CAATAGCCCTTGGATAAAAAAAAAA-FAM-3'.

Preparation of NP dimers stabilized with polyethylene glycol and functionalized with TAT peptide: A thiolated polyethylene glycol (PEG-SH, molecular weight: 5000)

solution was added to NP dimers with a PEG-to-NP ratio of 1000:1. After 15 min, the excess PEG was removed by centrifugation and washed with water three times. Then, the TAT peptide solution (5 mM) was added with a peptides-to-NP ratio of 1000:1. The solution was incubated for 24 h at ambient temperature followed by removal of the un-coupled peptide though centrifugation. NP dimers were centrifuged and washed with water three times.

Quantitation of number of TAT peptides per NP: The number of TAT peptide that are conjugated to the NP can be measured using the following procedure.

(1) Label TAT peptide with FAM fluorescent dyes using a standard labeling kit.

(2) Then, determine a linear calibration curve that relates the concentration of a fluorescently labeled TAT peptide and fluorescence intensity.

(3) After attachment of FAM labeled peptides to NP dimers with a peptides-to-NP ratio of 1000:1, centrifuge NP dimers and measure fluorescence signal from the supernatant at the 490 nm excitation/518 nm emission wavelengths of the FAM that were used to tag TAT peptide.

(4) Use the calibration curves to determine the concentration of TAT peptide in the supernatant. Subtract these values from the initial concentrations to obtain the amount of TAT peptide that is attached to NP dimers.

(5) To measure the initial concentrations, prepare TAT peptide solutions at exactly the same concentrations as in the reaction mixture with Au NP dimers.

NP dimers coated with PS-PAA were prepared when 600 μL dimethylformamide (DMF) solution of the polymer was added into the TAT-modified NP dimer dispersions, followed by 2-naphthalenethiol in DMF (20 μL, 3 mg/mL), and 80 μL PS_{15000}-*b*-PAA_{4300} (8 mg/mL in DMF) with stirring in accord with description by Wang *et al* in Ref.[3]

NRs and NR dimers coated with PS-PAA: Dispersions of NR and NR dimers in CTAB solution were centrifuged and washed with water three times to remove the free CTAB in solutions. 670 μL DMF, 80 μL PS_{15000}-*b*-PAA_{4300} (8 mg/mL in DMF), 2 μL of a 5 mM PEG-SH solution (molecular weight: 1000) and 120 μL 1-octadecanethiol (2 mM) were mixed, then 150 μL NR or NR dimer with 100 μL water were added to the mixture with stirring. Next, 200 μL of the mixture was washed with 900 μL water three times and coupled with TAT peptide with a peptide-to-NR ratio of 1000:1.

The sequences of the TAT peptide is YGRKKRRQRRRC.

***In vitro* cytotoxicity evaluation:** HeLa cells were cultured in a 96-well plate at a density of 10^5 cells per well. After incubation for 24 h at 37°C , dispersions of NP and NP dimers with concentrations of 1, 5 and 10 nM and NR@PS-PAA and NR dimer@ PS-PAA with concentrations of 1, 2 and 5 nM were dispersed into the medium, respectively. After 24 h incubation, the media was replaced and cells were treated with 3-(4,5-dimethyl-2-thiazolyl)-2,5-diphenyl-2-H-tetrazolium bromide (MTT) for 4 h.

Absorption was measured on a microplate reader at 450 nm. The flow cytometry was measured by Becton Dickinson FACS Calibur. The HeLa cells were seeded in a 6-well plate with a density of 10^5 cells per well. The NP dimers (10 nM) and NR dimers with PS-PAA shells (5 nM) were co-cultivated with HeLa cells for 2, 8, 12, 18, 24 and 48 h at 37 ºC, respectively. After the co-cultivation, the cells were removed by the standard trypsin lift-up procedure and washed with PBS three times. Then, Annexin V-FITC and propidium iodide (PI) solutions were added in the dark. The fluorescence was quantified by BD FACS-Calibur. All data was analyzed using 10,000 cells by FlowJo.

***In vitro* photodynamic therapy testing** was carried out for particles and their dimers modified with NH_2-PEG-SH instead of PEG-SH as in the previous protocol, with a PEG-to-NP ratio of 1000:1. Then, the well-known photodynamic therapy agent photosensitizer protoporphyrin IX (PpIX) was conjugated with NP dimer though *N*-(3-dimethylaminopropyl)-*N*'-ethylcarbodiimide (EDC) and *N*-hydroxysuccinimide (NHS) method. Subsequently, the TAT peptide solution was added to the NP dimer-PpIX dispersions or NP-PpIX dispersions with a peptide-to-NP ratio of 1000:1.

HeLa cells were seeded in six-well plates with a density of 10^5 cells per well. After 24 h incubation at 37 ºC, NP dimer-PpIX, NP-PpIX and PpIX were added to the cellular medium and the cells were incubated for additional 18 h. The media was removed and cells were washed with PBS three times. Afterwards, three groups of HeLa cells incubated with NP dimers-PpIX were irradiated by left circularly polarized light (LCP),

right circularly polarized light (RCP) and linearly polarized light (LP) for 30 min at 532 nm, respectively. Additionally, HeLa cells incubated with NP-PpIX and PpIX were irradiated by LP for 30 min at 532 nm. Cells without NP/NR addition in media were used as control. Cell viability was measured by MTT assay after different treatment, and further confirmed by a live/dead cell vitality assay kit (Invitrogen) by laser scanning confocal microscopy (Carl Zeiss LSM710). All the corresponding experimental series were replicated at least three times.

Quantification ***of ex-vivo singlet oxygen generation*** (SOG) was accomplished based on fluorescence of the singlet oxygen sensor green (SOSG). Briefly, 0.2 mL samples and 0.04 mL of 50 μM SOSG were mixed in black 96-well plates. After irradiation with the related polarized light (5 mW/cm^2 light at 532 nm for NP dimers and 5 mW/cm^2 light at 660 nm for NR dimers, respectively) for various time (0, 2, 4, 6, 8 and 10 min) and then the oxidized SOSG was quantified by measuring the fluorescence intensity (excited at 504 nm and measured at 525 nm) using a microplate reader. The experiments for each group were run in triplicate.

DNA bridges between particles*:* We choose the thiolated DNA anchors containing 10 adenines as a part of dsDNA (double stranded DNA) bridge because methylamine groups in each adenine are strongly adsorbed onto the surface of NPs. This is a significant addition to thiol-gold bond increasing the robustness of the construct.

Consequently, this segment does not extend away from the surface and contributes appreciably to the bridge length.

Park *et al*[4] found that for 40 base pairs and 22 free bases between two NPs, the interparticle distance was measured to be 27.9 nm, which constitutes 20-25% compression of DNA length. In this work, the length of DNA bridging the NPs and NRs contains 20 free bases and 15 base pairs-bridged can be calculated to be 7.3 nm in length based on the free-DNA conformation established by Gore *et al*.[5] However, the compression of DNA chains in the vicinity of gold NPs shortens its length. Based on the cryo-transmission electron microscopy (cryo-TEM) tomography, it was calculated to be 5.4±0.2 nm and 8.8±0.1 nm for the dimer outside and inside the cells, respectively.

The reduction of the DNA length in the vicinity of the gold surface can also be inferred from molecular dynamics simulation[6]. The dsDNA adopts a shorter B-form DNA conformation when linking two gold NPs in accord with data from Park et al [4] cited above. Using the data from Lee et al [6], we calculated the length of the linker DNA bridge in our assemblies to be 7.1 nm (i.e. 15 base pairs is 5.1 nm in length, 20 free bases is ~2 nm). For the calculation of spring elastic energy, the single stranded DNA is expected to be embedded in the PEG/TAT layer and thus is largely is immobile. Thus, the unstrained length of dsDNA of 5.1 nm is the base of the elastic energy estimates.

Electrostatic forces between two Au NPs or NRs: An expression for the electrostatic force between two gold elongated NPs at a separation distance *h* with a

dihedral angle θ is derived in this section. For brevity, we shall refer to both types of NPs as NRs. In order to simplify the derivation, several assumptions were made to arrive to order of magnitude estimates for W_{el} which are as follows:

(1) Each of the gold NRs are assumed to be a line of charges whose line charge density σ_{line} is approximated by charge density conversion from the experimentally obtained ζ-potential.

(2) The separation distance is defined along the orthogonal axis between two parallel planes, each plane encompassing the line charges. Therefore, the separation distance remains constant when the dihedral angle changes between the NRs and the derivation simplifies to 2D geometry, as shown in **Supplementary Figure 31**.

Line charge 1 (labeled NP/NR #1, with arc length coordinate $s_1,\ 0 \leq s_1 \leq l$) is set parallel to the x-axis and forms a dihedral angle θ with Line charge 2 (labeled NP/NR #2, with arc length coordinate $s_2,\ 0 \leq s_2 \leq l$). Importantly, the initial point of Line charge 1 contacts the y-axis and that of Line charge 2 contacts the x-axis. The electrostatic force between the differential charge elements of each NR, dq_1 and dq_2, is

(Supplementary Eq. 1)

$$\vec{F} = \frac{1}{4\pi\varepsilon_o\varepsilon_r}\int_0^l\int_0^l \frac{dq_1 dq_2}{r^3}\vec{r}$$

Here $\vec{r}$ is the displacement vector from dx_1 to dx_2 and r is its magnitude, ε_0 is the electrical permittivity of a vacuum, and ε_r is the dielectric constant of water. The differential charge elements are $dq_1 = \zeta ds_1$ and $dq_2 = \zeta ds_2$, products of a uniform

charge density (charge per unit length) ζ and differential arc lengths ds_1 and ds_2.

We now express $\vec{r}$ in terms of s_1 and s_2. In **Supplementary Figure 31** x_{2a} represents the location where the initial tip of Line charge 2 is projected onto the *x*-axis and x_{2b} represents the projection onto the *x*-axis of s_2, the arc length distance along Line charge 2.They can be expressed as follows:

$$x_{2a} = s \tan \gamma \qquad \text{(Supplementary Eq. 2)}$$

$$x_{2b} = s_2 \cos \theta, \qquad \text{(Supplementary Eq. 3)}$$

where $= {}^{l}/_{2} \sin \theta$, $\gamma = {}^{\theta}/_{2}$ as $2\alpha + \theta = 180^o$ (isosceles triangle), $\gamma = \alpha - \beta$ and $\beta = 90^o - \theta$. Hence, the *x*-coordinate of the point at arc length distance s_2 along Line charge 2 is

$$x_2 = x_{2a} + x_{2b} = \frac{l}{2} \sin \theta \tan \frac{\theta}{2} + s_2 \cos \theta \qquad \text{(Supplementary Eq. 4)}$$

The *y*-coordinate of the same point is expressed as follows via simple trigonometry:

$$y_2 = -s + s_2 \sin \theta = (s_2 - {}^{l}/_{2}) \sin \theta. \qquad \text{(Supplementary Eq. 5)}$$

The *z*-component of $\vec{r}$ (that is aligned with h coordinate in Supplementary Eq.1) remains constant at all arc lengths s_1 and s_2 along the nanorods at the experimentally

(Supplementary Eq. 6)

found separation distance$h$. Therefore, the displacement vector expressed in terms of s_1 and s_2 is as follows:

$$\vec{r} = \left(\frac{l}{2}\sin\theta\tan\frac{\theta}{2} + s_2\cos\theta - s_1\right)\hat{x} + ((s_2 - \frac{l}{2})\sin\theta)\,\hat{y} + h\hat{z}$$

Substituting and integrating over the length of each line charges, the total electrostatic force between Line charges 1 and 2 is obtained as follows:

$$\vec{F} = A\int_0^l\int_0^l \frac{\left[\left(\frac{l}{2}\sin\theta\tan\frac{\theta}{2} + s_2\cos\theta - s_1\right)\hat{x} + ((s_2 - \frac{l}{2})\sin\theta)\,\hat{y} + h\hat{z}\right]}{\left(\frac{l}{2}\sin\theta\tan\frac{\theta}{2} + s_1\cos\theta - s_1)^2 + ((s_2 - \frac{l}{2})\sin\theta)^2 + h^2\right)^{3/2}}\,ds_2 ds_1 \quad \text{(Supplementary Eq. 7)}$$

, where

$$\mathrm{A} = \frac{\sigma_{line}^2}{4\pi\varepsilon_0\varepsilon_r} \quad \text{(Supplementary Eq. 8)}$$

$$\sigma_{\mathrm{line}} \approx \frac{2\varepsilon_r\varepsilon_o\kappa k_B T}{e}\sinh\left(\frac{e\zeta}{2k_B T}\right) \quad \text{(Supplementary Eq. 9),}$$

where k_B is the Boltzmann constant and T is the absolute temperature. For simplicity, 1-1 electrolytes are assumed in the surrounding media for charge density approximation from the ξ-potential measurements (Supplementary Eq. 9)[7]. Debye length, κ, is given by,

$$\kappa = \sqrt{\frac{1000 N_A e^2}{\varepsilon_r\varepsilon_0 k_B T}\sum_i M_i \times z_i^2} \quad \text{(Supplementary Eq. 10)}$$

where e is electric charge (in Coloumbs), N_A is Avogadro's number, and M_i and z_i are the molar concentration and valency of ions, respectively, where $z_i = 1$ is assumed. Supplementary Eqs. 9 and 10 can be used to calculate the surface charge density based on zeta-potential measured in the buffer, which represents the case of which is fairly well described by the DLVO theory especially for the extracellular case.

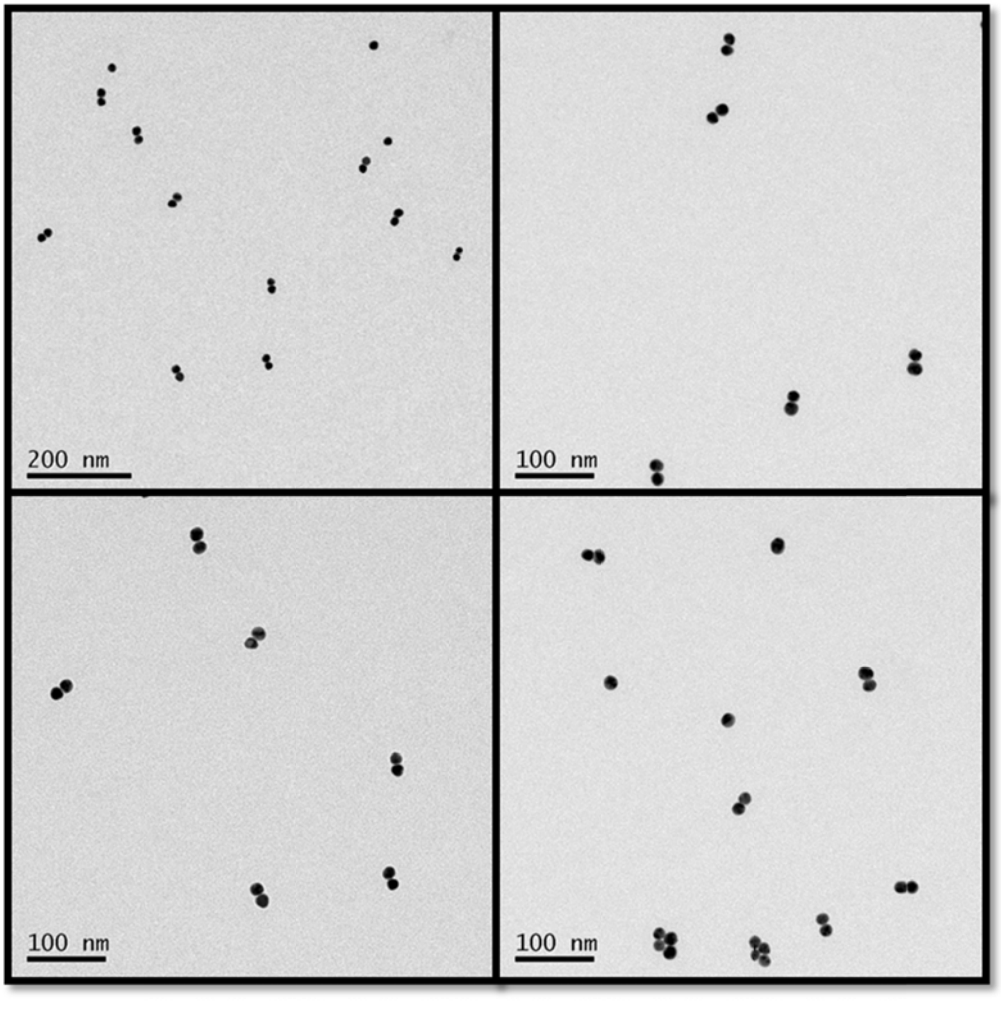

Supplementary Figure 1. TEM images of NP dimers in 0.01 M phosphate buffer (PB) at pH 7.4.

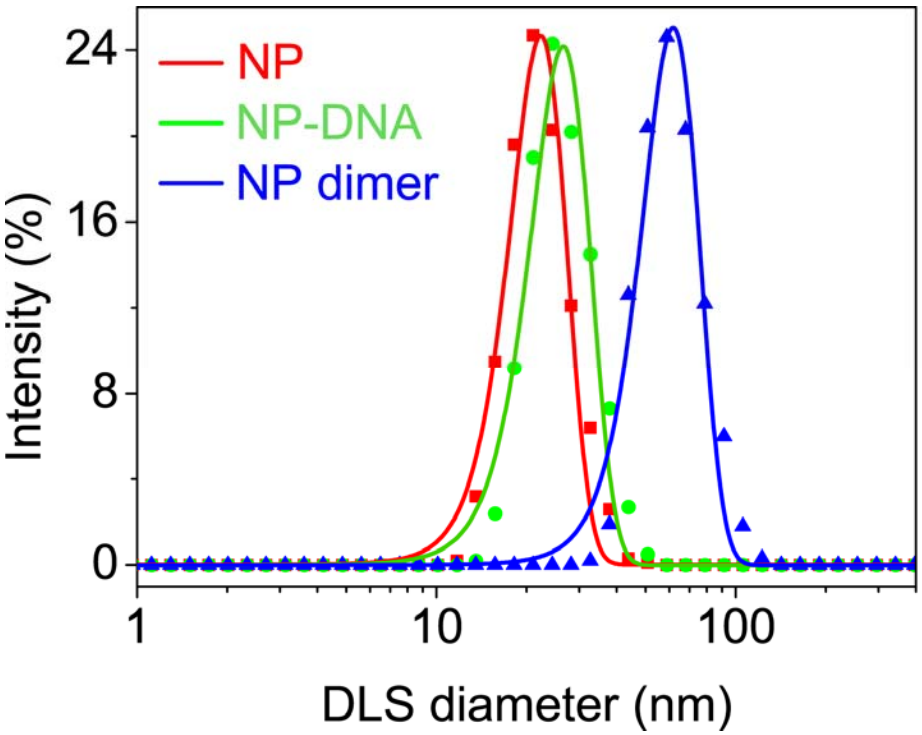

Supplementary Figure 2. Dynamic light scattering (DLS) spectrum of the NPs, NP-DNA conjugate, and DNA-bridged NP dimers.

Comment: The hydrodynamic diameter of the NPs were 22±3 nm, after assembly the diameter of dimer increased to 65±2 nm (**Supplementary Figure 2**). These values match well with the expected size of NP dimers.

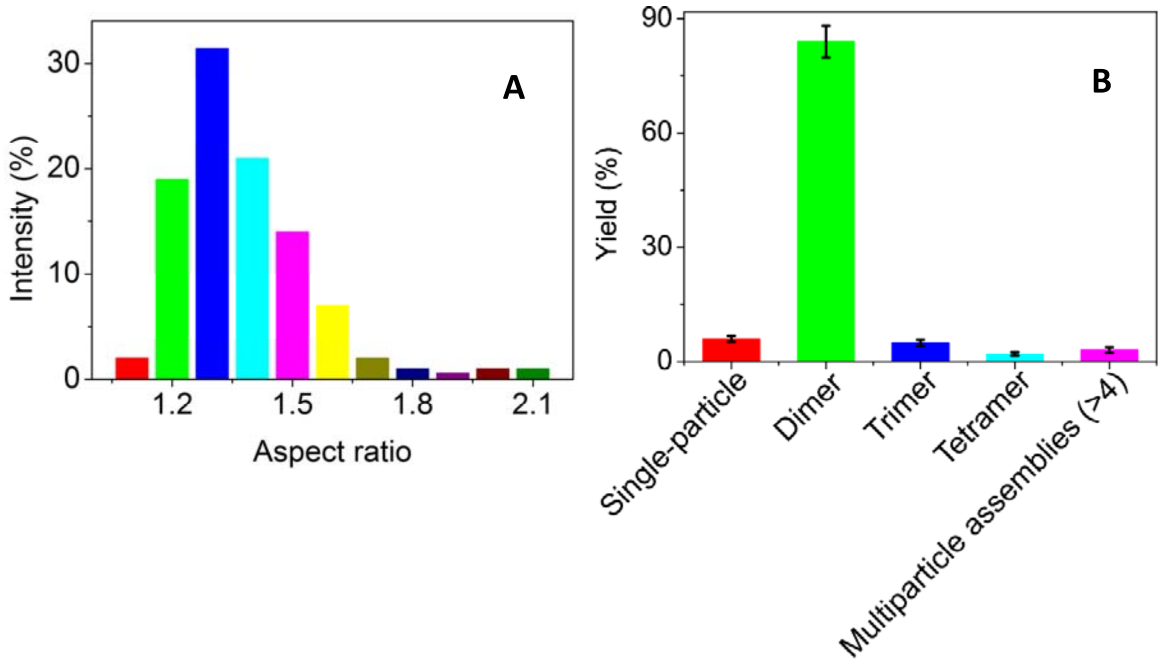

Supplementary Figure 3. (**A**) Statistical analysis of the aspect ratios of Au NPs determined by statistical analysis of 300 nanoparticles bright-field TEM; (**B**) Statistical analysis of different products in the reactions of dimer assembly. The yield of NP dimerization products was calculated using TEM images. For NP dimers of interest is was 85±4.2%. The error bars correspond to the standard error of the mean (n = 3).

Comments: The safety of exogenous DNA does not appear to be problematic to our systems because (a) the sequence of DNA was non-coding region (checked from NIH genetic sequence database) and could hardly alter cell behavior and (b) NPs prevent penetration of DNA into cellular nucleus or mitochondria where it can be integrated with the centromeres or sub-telomeric regions to cause mutation.[8]

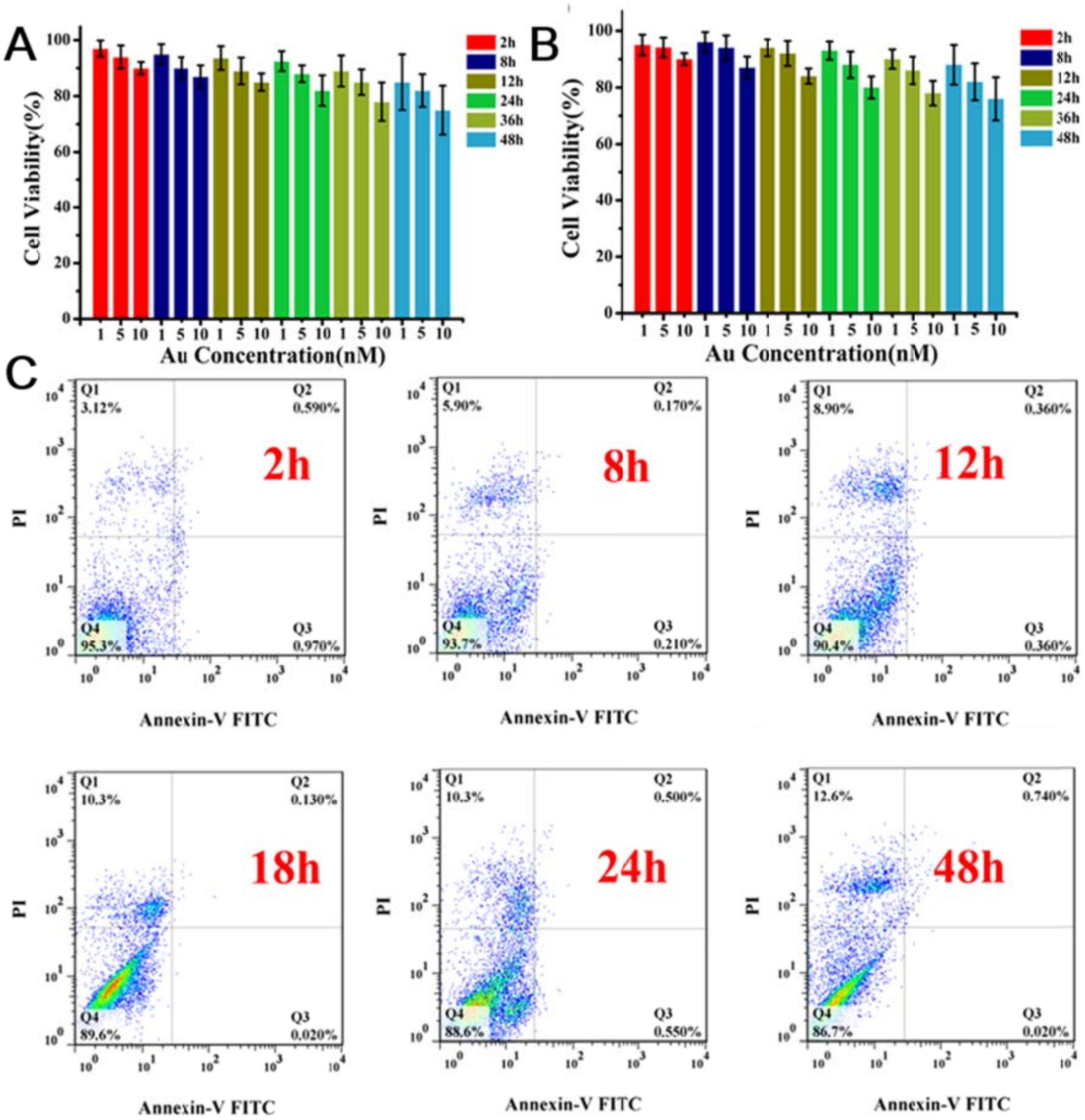

Supplementary Figure 4. Cell viability measured by MTT test for (**A**) individual NPs, (**B**) NP dimers. (**C**) Flow cytometry of HeLa cells with Annexin-V marker after incubation with NP dimers. The error bars correspond to the standard error of the mean (n = 3).

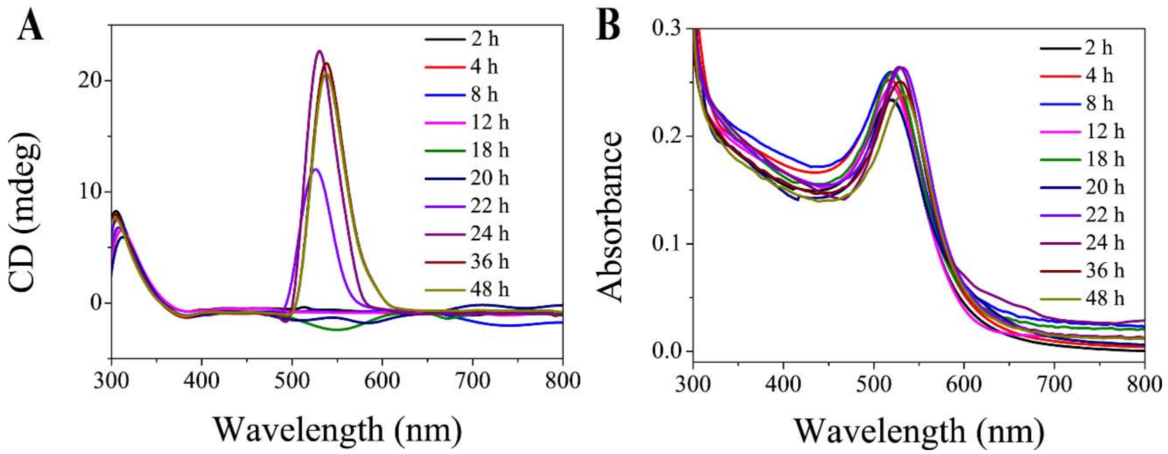

Supplementary Figure 5. CD (**A**) and UV-vis (**B**) spectra of NPs incubated with HeLa cells for different periods of time from 2 h to 48 h without removing the excess extracellular NPs.

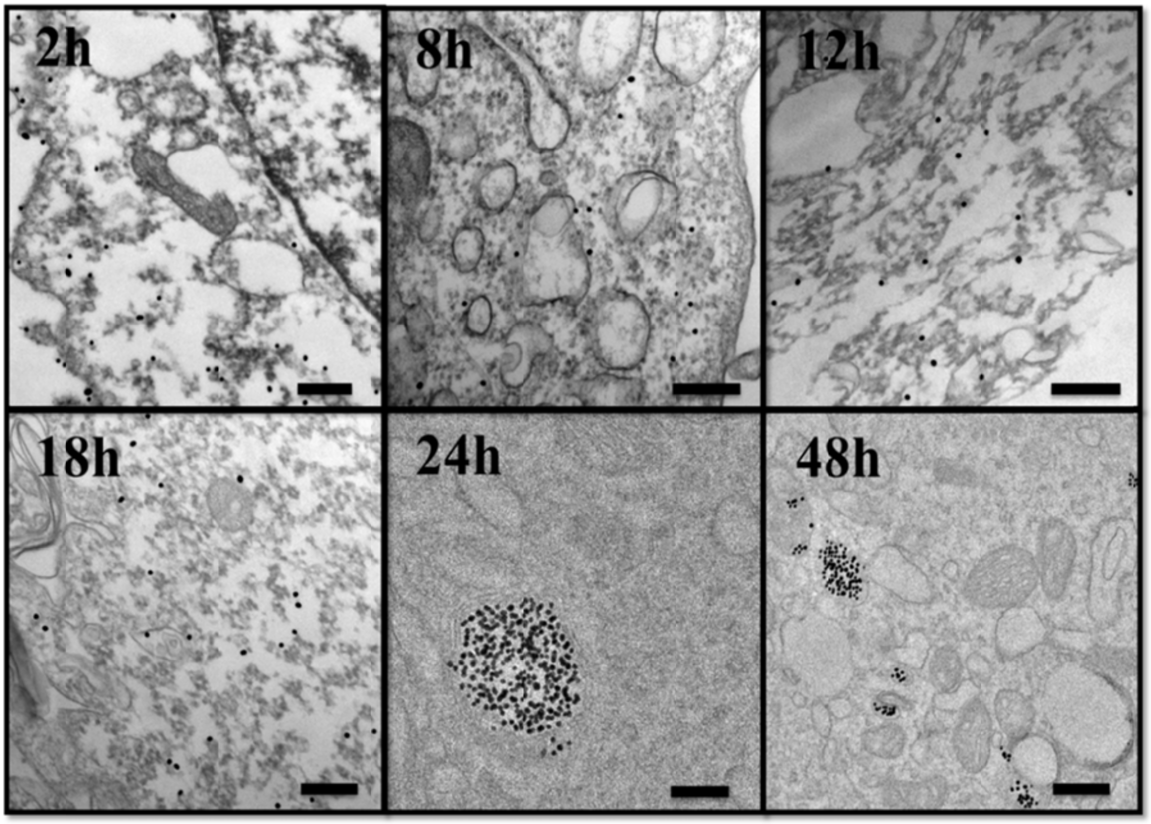

Supplementary Figure 6. Bio-TEM images of NPs in living cells with different incubation times from 2h to 48 h indicated in the micrographs. Scale bars are 200 nm.

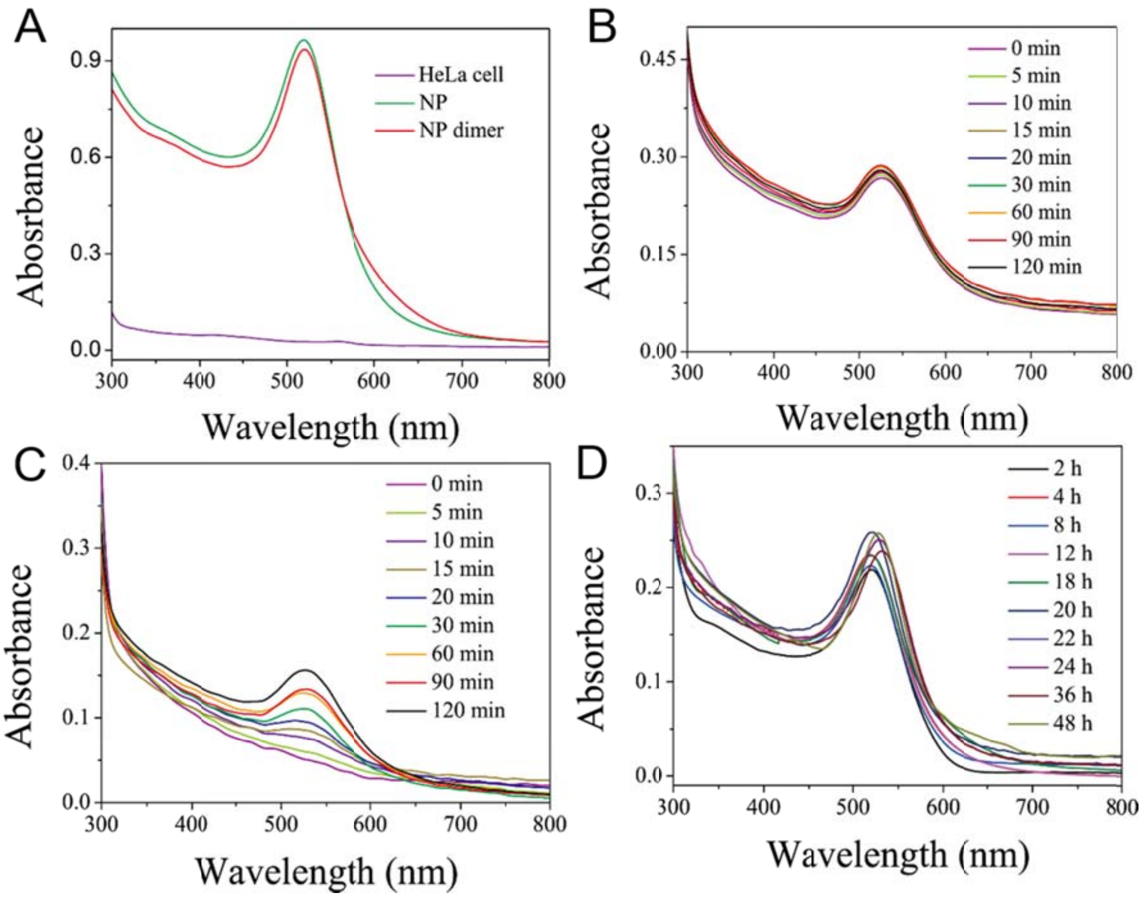

Supplementary Figure 7. Ultraviolet-visible (UV-vis) spectra of NP dimers in HeLa cell cultures. (**A**) UV-vis spectra of individual NPs, HeLa cells and NP dimers in the TBE buffer. (**B-C**) Temporal progression of UV-vis spectra of NP dimers incubated with HeLa cells for 2 h and measured before (**B**) and after (**C**) the removal of extracellular excess of NP dimers. (**D**) Temporal progression of UV-vis spectra of NP dimers incubated with HeLa cells for 48 h after removal of the excess NP dimers from the outside of the cells.

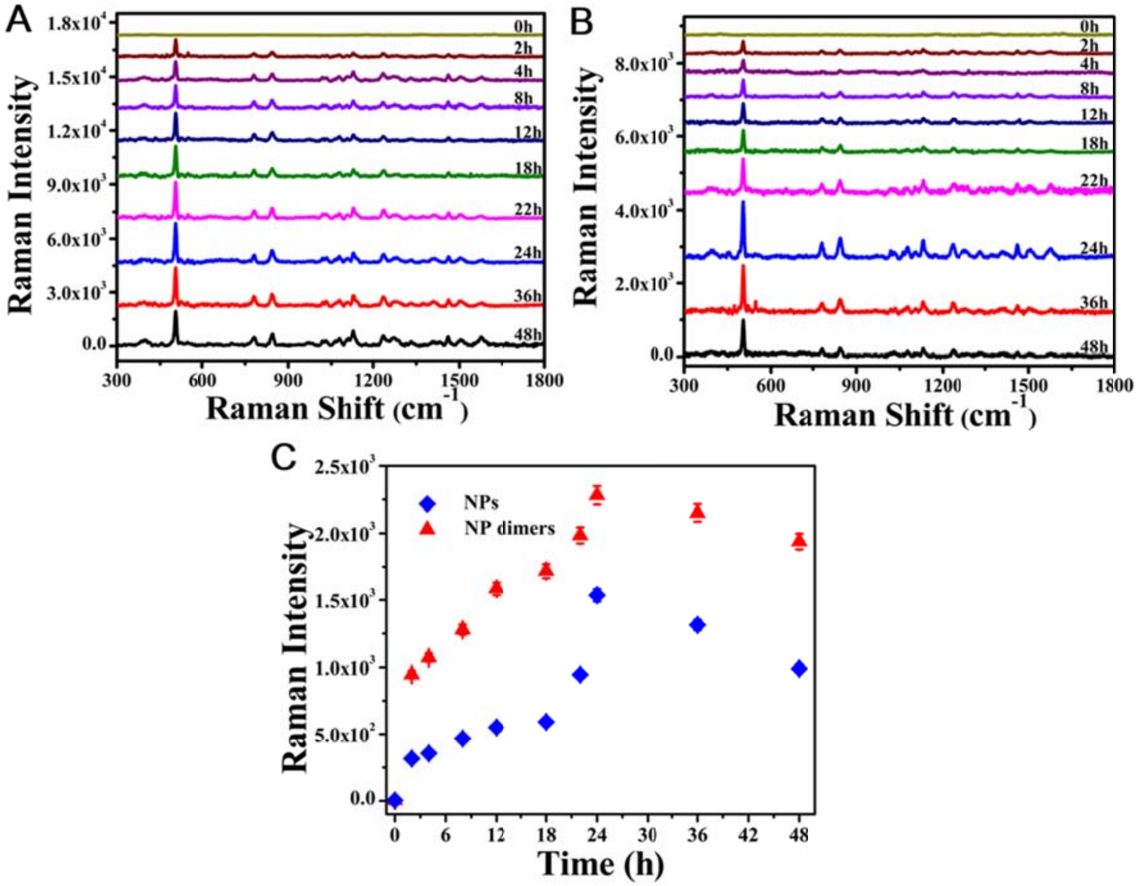

Supplementary Figure 8. Effect of transmembrane transport of NPs and NP dimers on SERS spectra. (**A**) Surface-enhanced Raman scattering (SERS) spectra of NP dimers and (**B**) NP in living HeLa cells. (**C**) SERS intensity progression for NP dimers and NPs at 471 cm^{-1} in living HeLa cells. The error bars correspond to the standard error of the mean (n = 3).

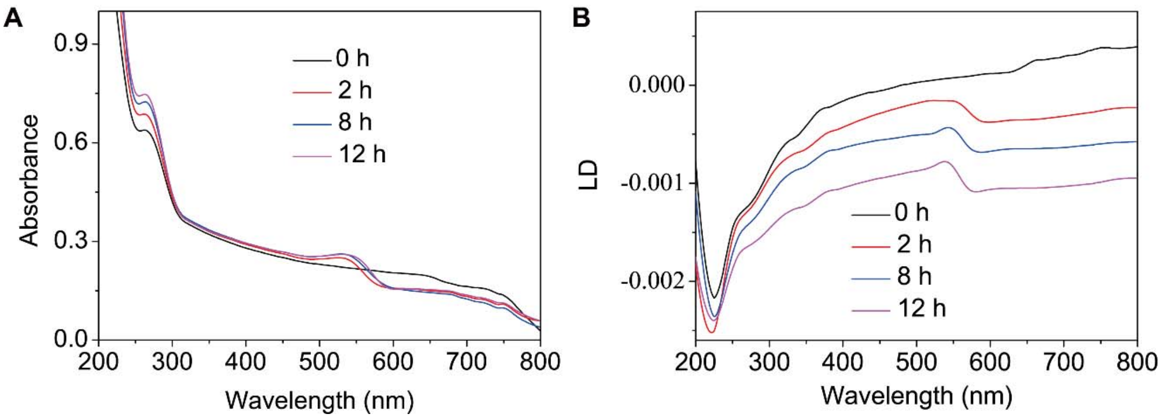

Supplementary Figure 9. Temporal progressions of linear dichroism (LD) and absorbance spectrum of NP dimers incubating with cells. Absorbance (**A**) and LD (**B**) spectra of NP dimers incubated with HeLa cells onto the glass over a period of 48 h.

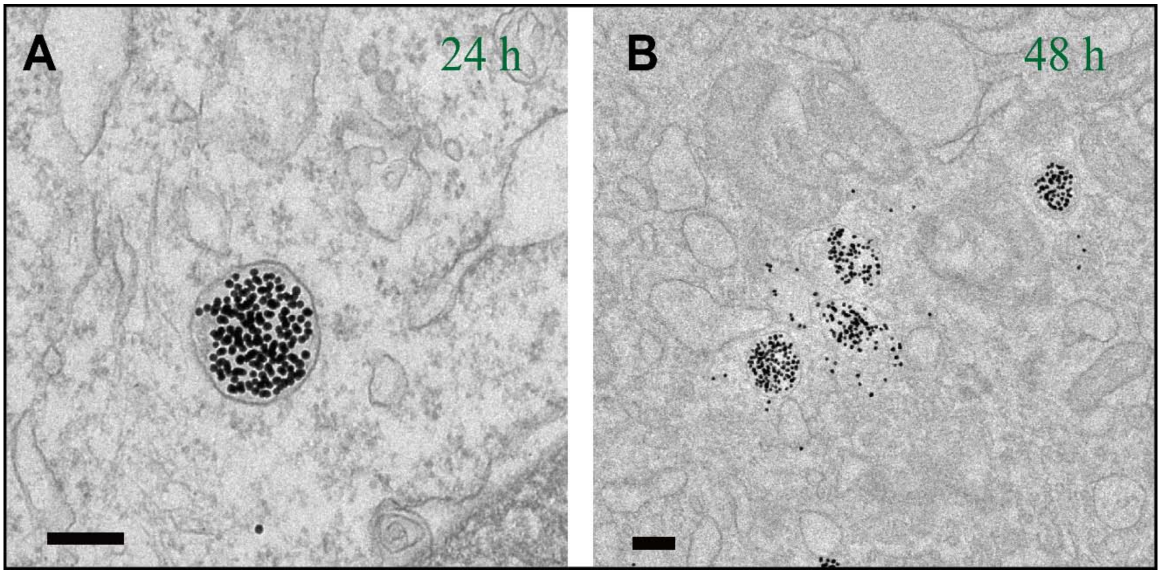

Supplementary Figure 10. Bio-TEM images of NP dimers in HeLa cells with (**A**) 24 h and (**B**) 48 h incubation times; Scale bars are 200 nm.

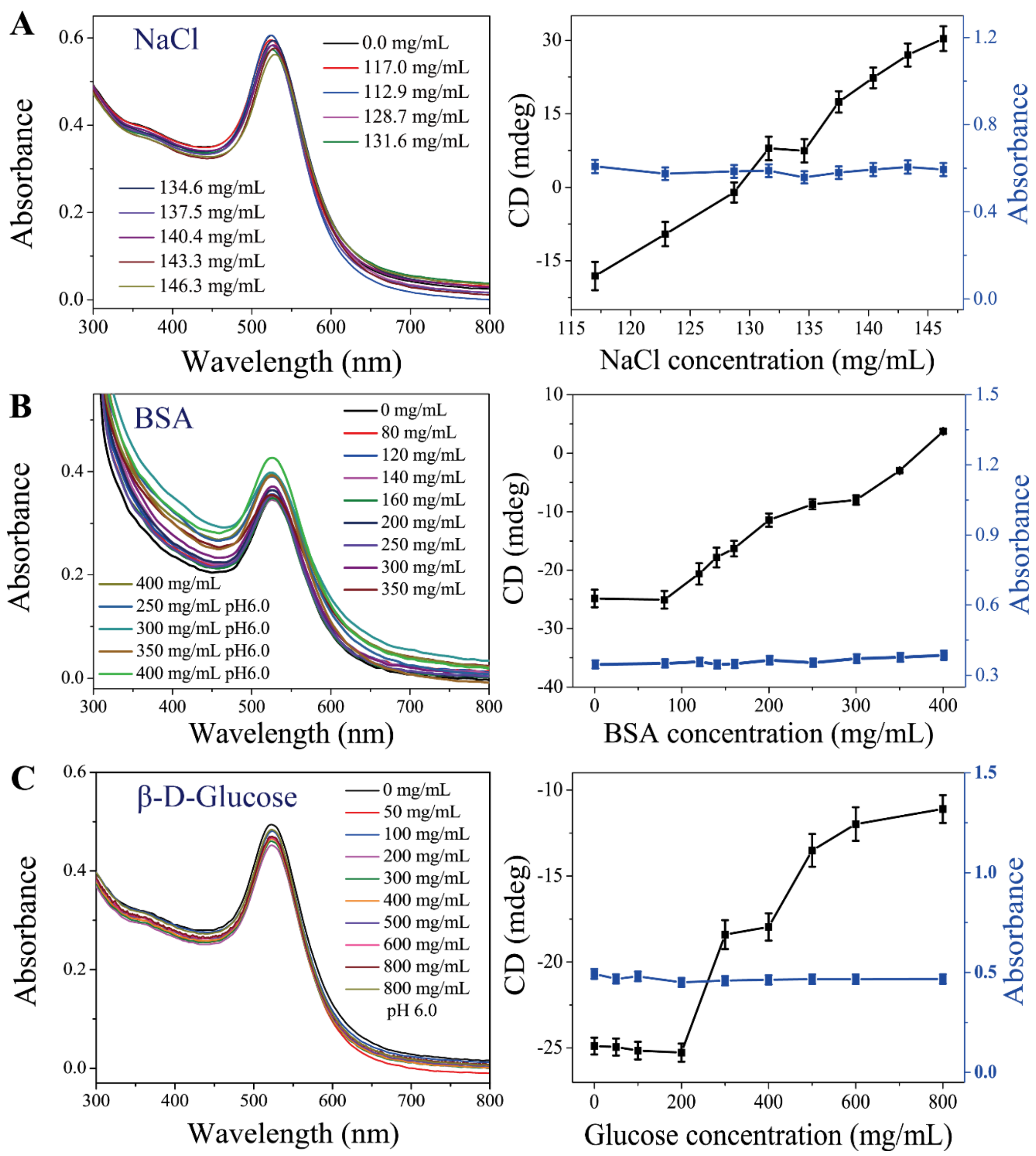

Supplementary Figure 11. Comparison of the concentration progressions for CD vs. UV-vis spectra of NP dimers in (**A**) NaCl, (**B**) BSA and (**C**) β-D-glucose solution. The error bars correspond to the standard error of the mean (n = 3).

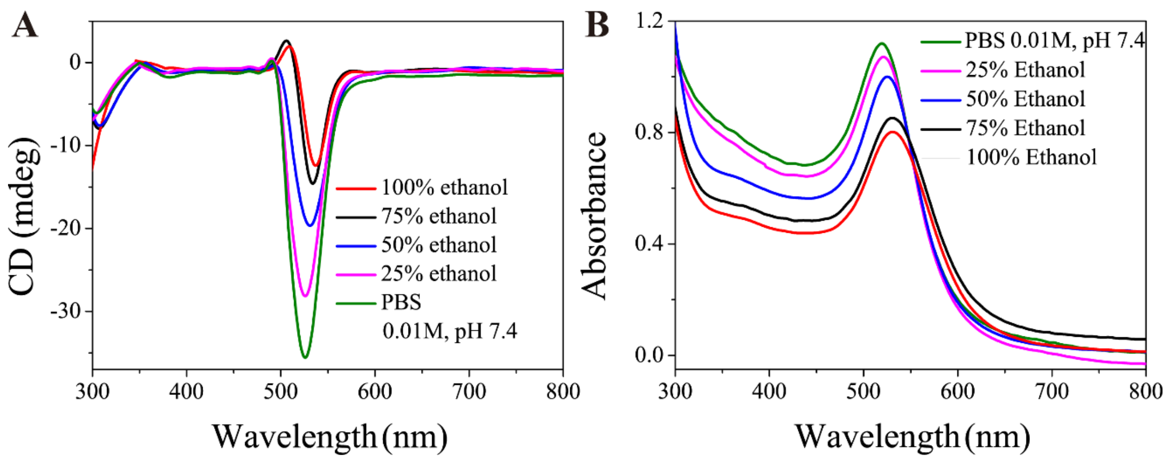

Supplementary Figure 12. (**A**) Circular dichroism **(**CD) and (**B**) ultraviolet–visible (UV-vis) spectra of NP dimers in ethanol-buffer mixtures with various volume fractions indicated in the graphs.

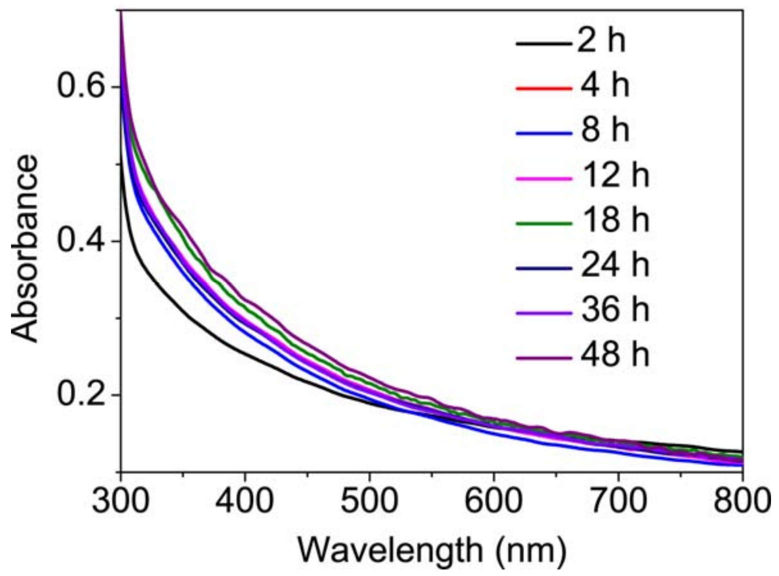

Supplementary Figure 13. The UV-vis spectrum of NP dimer without cell-penetrate peptides modification were incubated with cell for 48h. No internalization was observed.

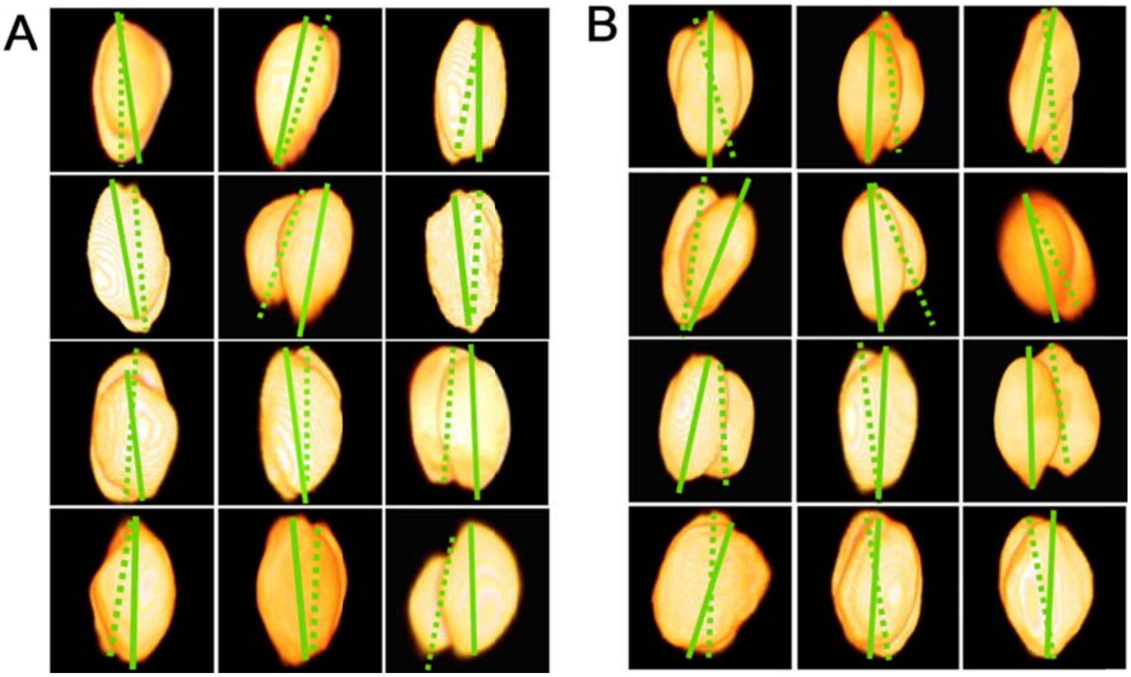

Supplementary Figure 14. TEM tomography images of selected NP dimers found in (**A**) in culture media and (**B**) in cytosol of HeLa cells. The appearance of higher-than-average anisotropy compared to bright filed TEM images is due to the 'missing wedge' problem associated with the limitation of the tilt angle to ±60°. Full statistical analysis of the aspect ratio and dihedral angles is given in **Supplementary Figures 3** and **16**.

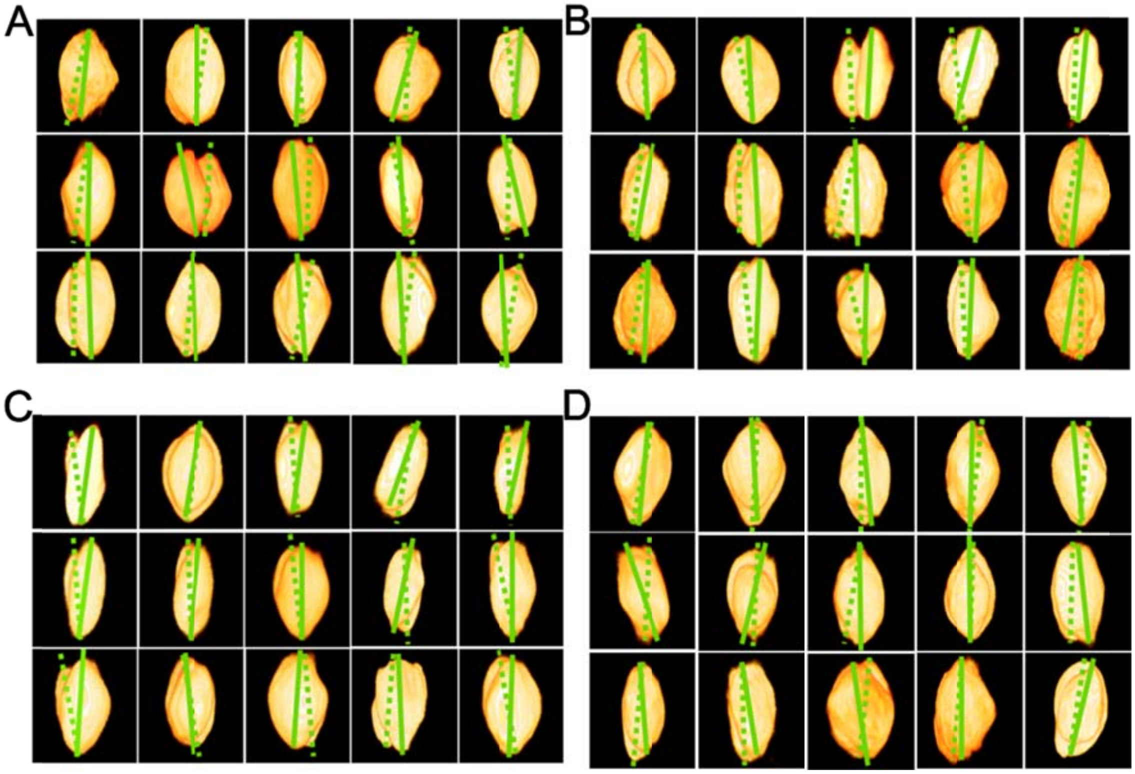

Supplementary Figure 15. TEM tomography images of selected NP dimers found in BSA solution (**A**) 200mg/mL (pH 7.4), (**B**) 400mg/mL (pH 7.4), (**C**) 400mg/mL (pH 6.0), (**D**) in glucose solution 800mg/mL (pH 7.4). The appearance of higher-than-average anisotropy compared to bright filed TEM images is due to the 'missing wedge' problem associated with the limitation of the tilt angle to ±60°. Full statistical analysis of the aspect ratio and dihedral angles is given in **Supplementary Figures 3** and **16**.

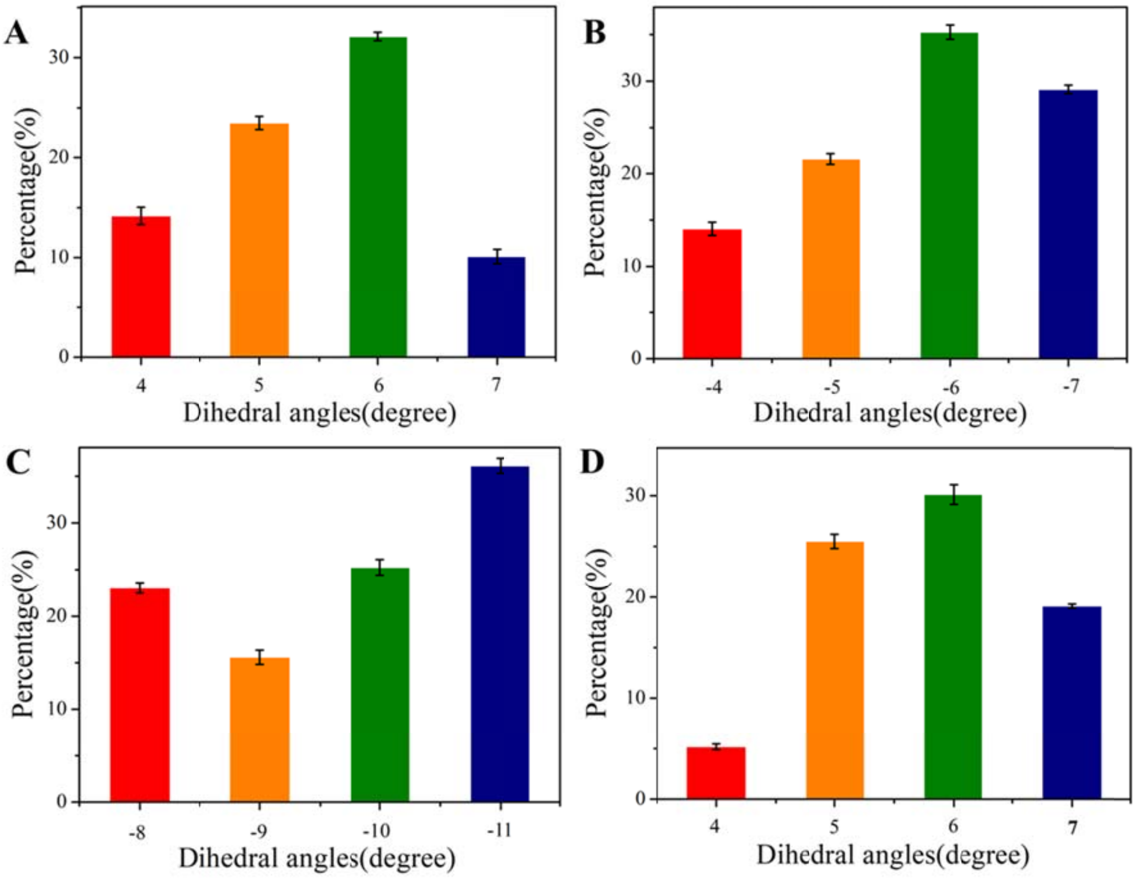

Supplementary Figure 16. Statistical analysis of dihedral angles θ in NP dimers in BSA solutions with concentrations of (**A**) 200 mg/mL (pH 7.4), (**B**) 400 mg/mL (pH 7.4), (**C**) 400 mg/mL (pH 6.0), (**D**) in glucose solution 800 mg/mL (pH 7.4). The statistical analysis of dihedral angles θ was obtained on the basis of measurement of geometry for 78, 76, 81 and 71 dimers from cryo-TEM tomography for the four groups above, respectively. The error bars correspond to the standard error of the mean (n = 3).

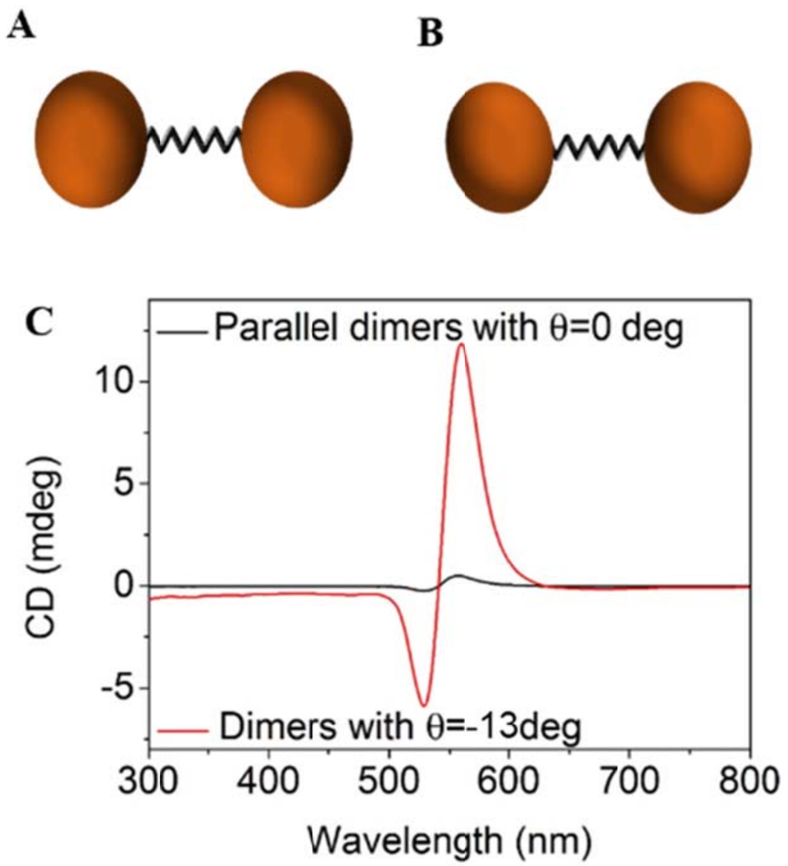

Supplementary Figure 17. Schematics (A, B) and calculated CD spectra (C) of NP dimers bridged by DNA-like organic helix characterized by dihedral angles $\theta = 0^o$ and $\theta = -13^o$, respectively.

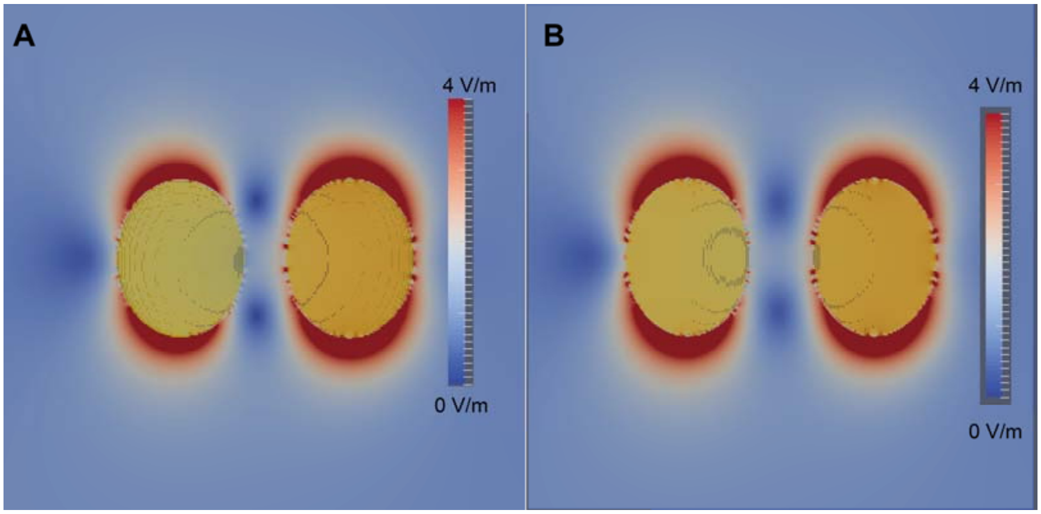

Supplementary Figure 18 | Simulation electromagnetic field strength for NP dimers outside (**A**) and inside (**B**) of cells. The calculated parameters (dihedral angle (*θ)* and gap distance) were -9.6 ^{o}, 5.4 nm for **A** and θ = -13°, 8.8 nm for **B**, respectively.

Supplementary Note: Let us specifically address the hypothesis for chirality reversal based on CD enhancement by hot-spots between NPs. It predicates that chiroptical activity in our system originates from the chiral molecules (DNA, TAT peptide, or components from the cellular milieu) placed in the hot-spots.[9–14] When the molecule in the hot spot changes, the resulting chroplasmonic activity may switch the polarity. However after careful consideration of the hot-spot hypothesis, we were not able to explain the observed experimental facts. Here are four primary reasons why we had to choose a different model for explaining chiroptical activity and, hence, chirality reversal.

1. The study by Zhang *et al*[15] considers the hot-spot mechanism in a NP dimer

bridged by DNA in details. The absorption spectra of chiral molecules and gold NPs appear to be similar to ours. At first glance, this is a convenient pathway to explain occurrence of chiroptical activity in DNA-NP constructs including the NP-dimers. However, after a closer examination, this is not the case. Our dimers represent a system which far from being optimal for hot spots. Zhang *et al*[15] calculated that the circular dichroism associated with the hot-spots has a maximum intensity when the interparticle gap is 0.5-1 nm and rapidly drops as the distance increases. The gaps between the NPs in our dimers are much greater approaching 10x difference with the optimal one calculated by Zhang *et al.*[15] At these distances hot spot enhancement of the "natural" circular dichroism of DNA is much less efficient than in their calculations. Therefore, it is difficult to claim the match with this experimental object.

2. Although absorption spectra are similar to ours, the CD spectra calculated by Zhang *et al*[15] are different than ours (e.g. **Figures 1B**) -- they have multiple spectral features that we do not have and vice versa despite the apparent similarity of the dimers in the cartoons. Potentially such differences could be attributed to the binding of specific components of intracellular media to DNA bridges or particles or formation of protein corona.[16–18] In turn, hot-spot enhanced CD could result in appearance of more complex spectral shapes as in Figure 2d from Zhang *et al*[15]. However, no spectra similar to those calculated by Zhang *et al*[15] could be observed (**Figure 1**). In fact, addition of biological components, for

instance BSA and β-D-glucose with opposite handedness of optical centers on sp^3 carbons, did not change the spectral position of chiroptical peaks in the plasmonic region (**Figure 1, F,H**).

3. Extensive theoretical and experimental studies indicate that the intensity of electrical field in the hot-spots is tracked by the intensity of the surface-enhanced Raman scattering,[19,20] *i.e.* the 'hotter' the hot spot, the stronger Raman scattering is. As shown in **Supplementary Figure 8**, the intensity of Raman scattering band at ~513 cm^{-1} reach the maximum at 24 h. If the hot-spots determine the chiroptical activity in our system, the circular dichroism should also reach the maximum at 24 h without the significant plasmonic band shift. However, this is not the case (**Figure 1E**).

4. A separate computational study of the potential contribution of hot-spots to chiroptical activity of the dimers was carried out specifically for the NP dimers employed in our study. Chiroptical and absorbance spectra were calculated with the full space integration (4π ster) for two NP dimers bridged by a DNA-like helix with a refractive index of 1.45 and a polarizability of 4.06×10^{-23} cm^3. These values are similar to those of DNA. The refractive index and polarizability of dsDNA used for bridging dimers is known to be 1.34 and 3.18×10^{-23} cm^3, respectively (5'-CAATAGCCCTTGGAT-3' and 5'-ATCCAAGGGCTATTG-3').[21–23] **Supplementary Figure 17** and **18** presents the results of these calculations for the two NP dimes. Their geometry was

identical except the dihedral angle θ being 0 deg for one model and -13 deg for the other (**Supplementary Figure 17**). The simulations reveal that in both cases hot spots from coupling of the plasmonic oscillations in the highly polarizable particles are indeed formed. The highest intensity of the electrical field between the NPs for the parallel dimer with θ = 0 deg is 1.2 V/m and for twisted dimer with θ = -13 deg is 0.6 V/m, which are values characteristic of quite “cold” hot-spots. The maxima of the CD spectra for the parallel dimer with θ = 0 deg and for twisted dimer with θ = -13 deg were 1.3 mdeg and 12 mdeg, respectively. Although hot-spots are present, their role in chiroptical activity of the dimer is less significant (~10x) than geometry of the dimer.

Additional secondary reasons and experimental facts that do not bode well with hot spot mechanism in the particular case of NP assemblies studied in this work include the following.

1. Data in **Figure 1G** show that chiroptical activity of DNA-bridged dimer in absence of all other biological components changes the sign of polarization rotation in response to the concentration of sodium chloride, NaCl. The effect is strong - polarization rotation switches from -25 mdeg when [NaCl] = 0 mg/mL to +30 mdeg when to [NaCl] = 146.3 mg/mL. NaCl is not chiral and the DNA bridge retains its original helicity. Thus, the change of the hot-spot intensity or the appearance of the new chiral molecules in the interparticle gap whose circular dichroism is enhanced

by the NP pair cannot explain the reversal of sign of polarization rotation in **Figure 1**.

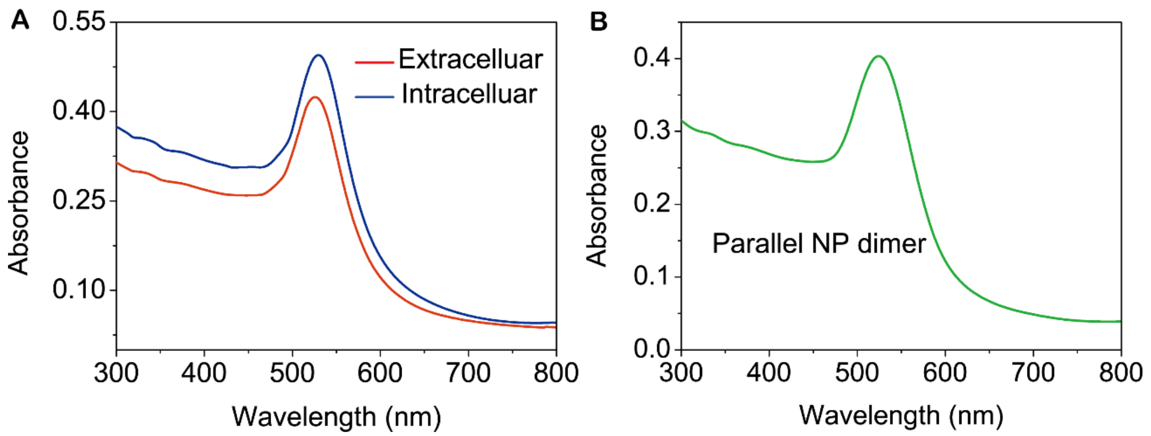

Supplementary Figure 19. FDTD-simulated UV-vis spectra of (A) NP dimers inside and outside of cells with the dimer geometry inferred from cryo-TEM tomography and statistical analysis of the dihedral angles θ from **Supplementary Figure 16** and (B) parallel NP dimers with $\theta = 0$ deg.

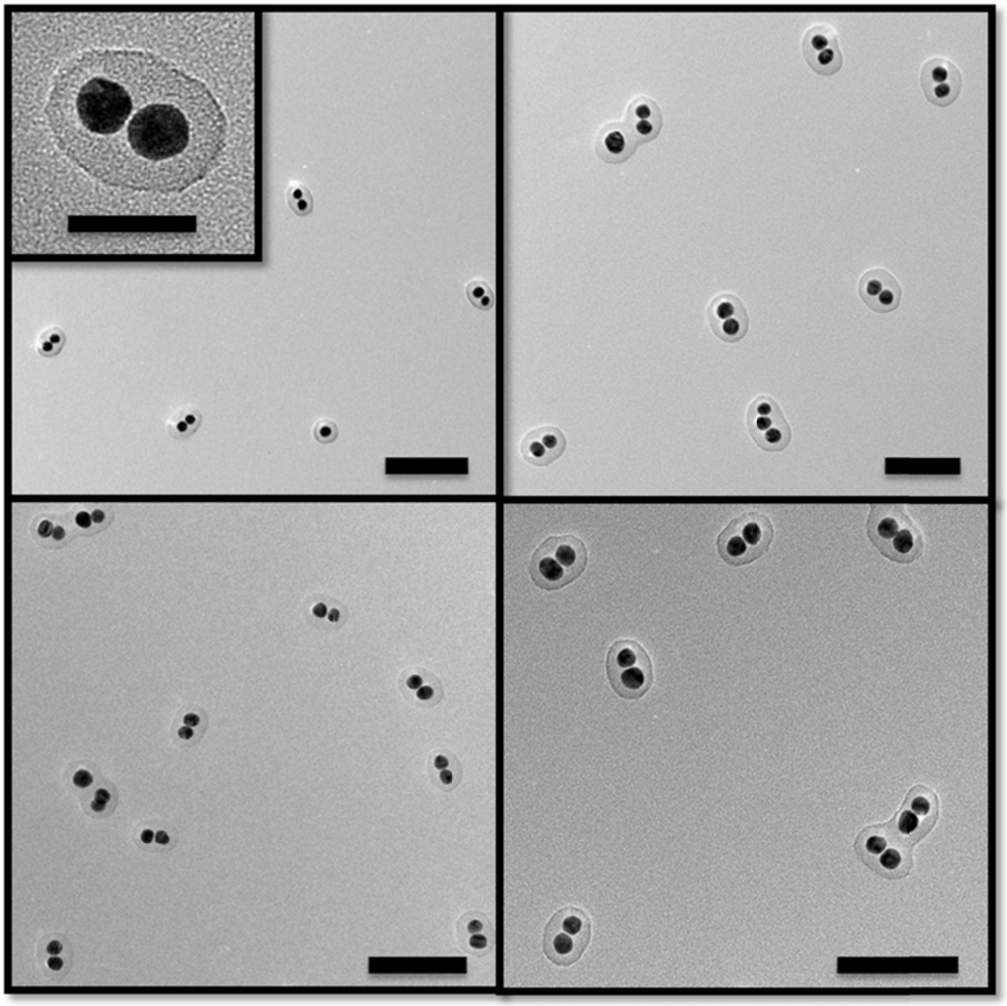

Supplementary Figure 20. TEM images of NP dimers with PS-PAA shells. Scale bars are 100 nm.

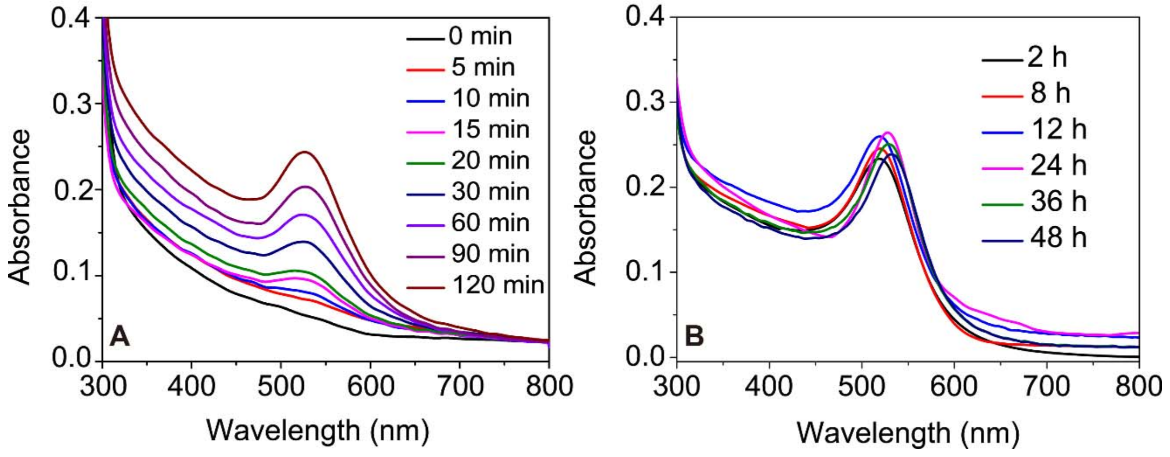

Supplementary Figure 21. Temporal progression of the UV-vis spectra for NP dimers with PS-PAA shells incubated with HeLa cells after the removal of extracellular excess of dimers, with different incubation times ranging from 0 h to 2 h (**A**) and from 2 h to 48 h (**B**).

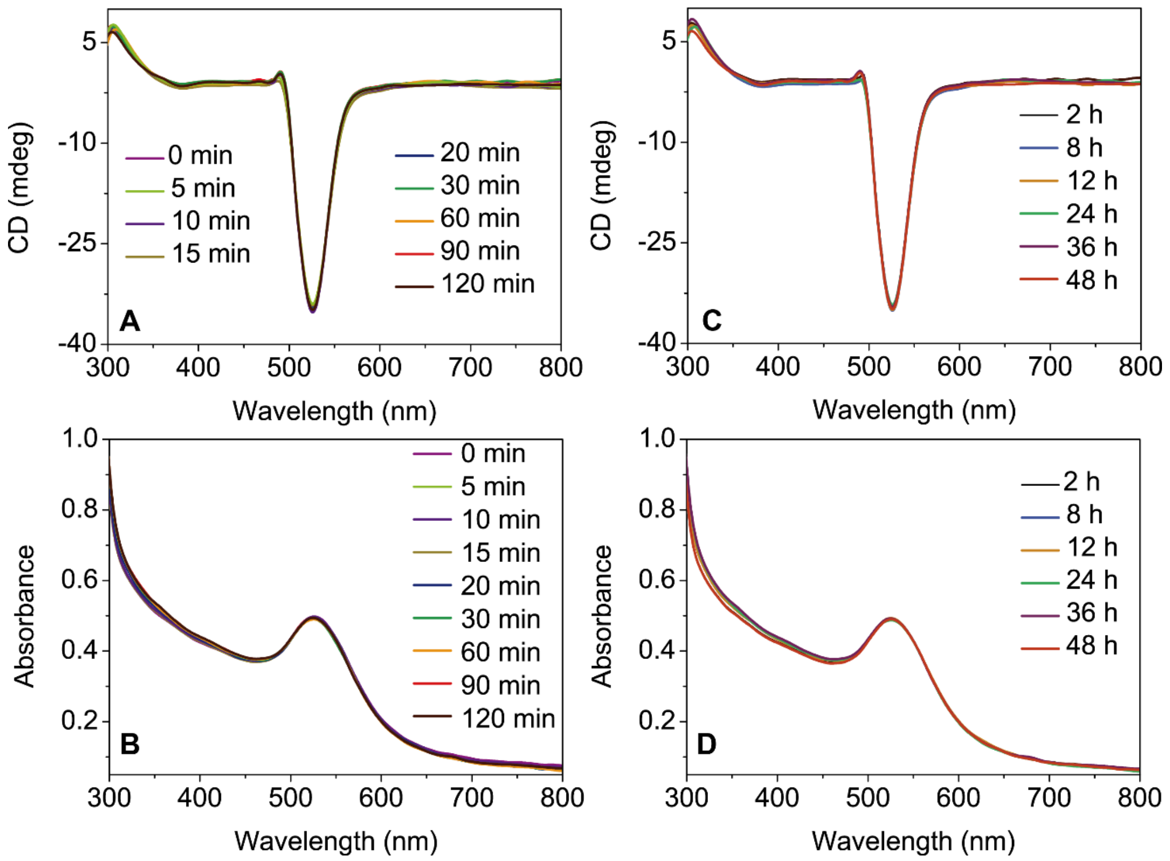

Supplementary Figure 22. Temporal progression of the CD (**A,C**) and UV-vis (**B,D**) spectra of NP dimers with the PS-PAA shells in HeLa cells without the removal of extracellular excess of dimers after incubation times ranging from 0 min to 2 h (**A,B**) and from 2 h to 48 h (**C,D**).

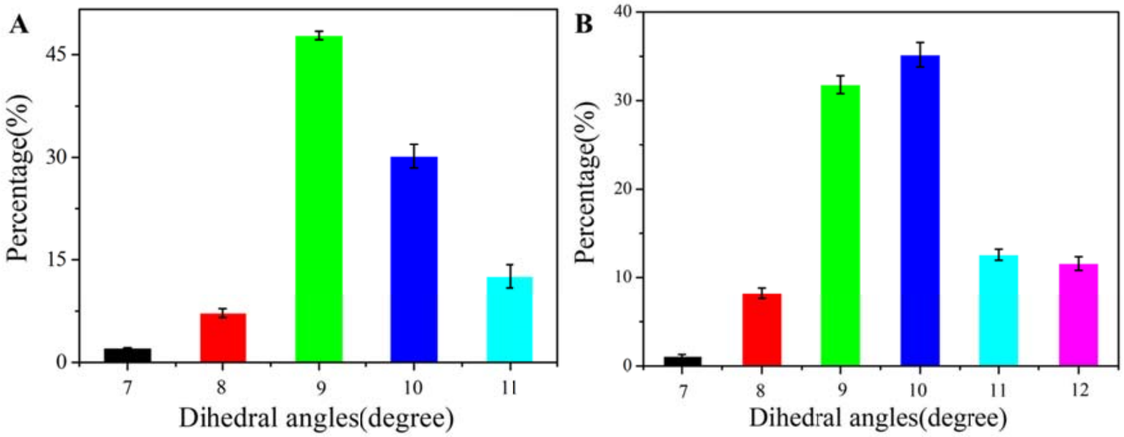

Supplementary Figure 23. Statistical analysis of the dihedral angles for NP dimers before (A) and after (B) encapsulation by PS-PAA. The error bars correspond to the standard error of the mean (n = 3).

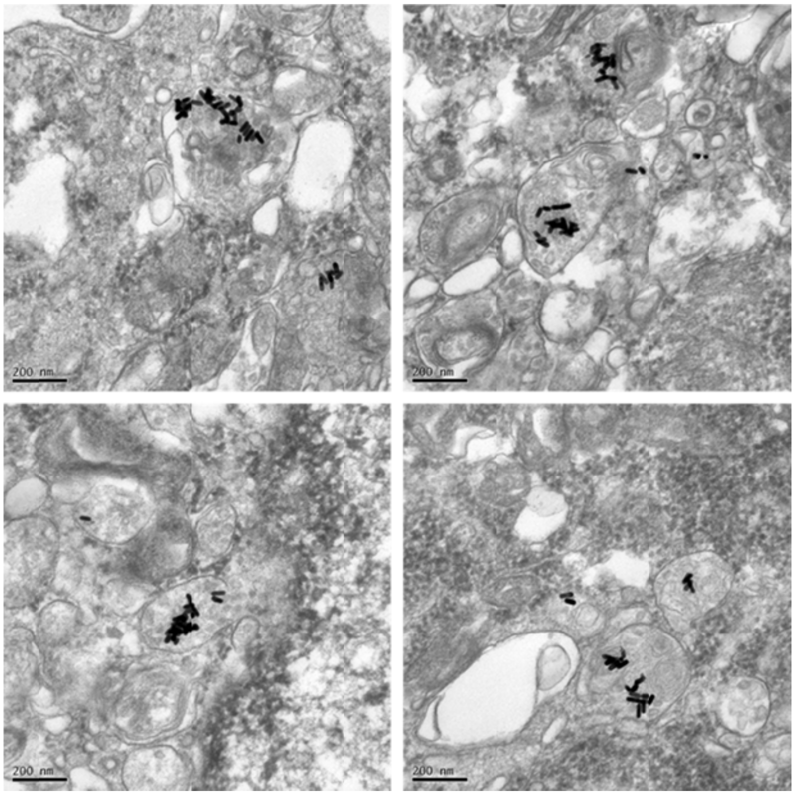

Supplementary Figure 24. The bio-TEM image of NR dimer after incubation with HeLa cells for 8h without PS-PAA coating. Scale bar 200nm.

Comment: Unconstrained gold NRs (no PS-PAA coating) are the natural choice to construct dimers with the angled configuration and observe chirality reversal upon transport into cells. The unconstrained NR dimers carrying PEG surface ligands and cell-penetrate peptides were made similarly to those from NPs. However, incubation with HeLa cells showed that these NR dimers agglomerate in cytosol. From the bio-TEM in **Supplementary Figure 24**, we can see that the NR dimers in cell inside the cells exist as aggregates rather than individual constructs as the case with NPs (compare with

Supplementary Figure 6). The formation of the aggregates makes the interpretation of CD spectra more difficult.

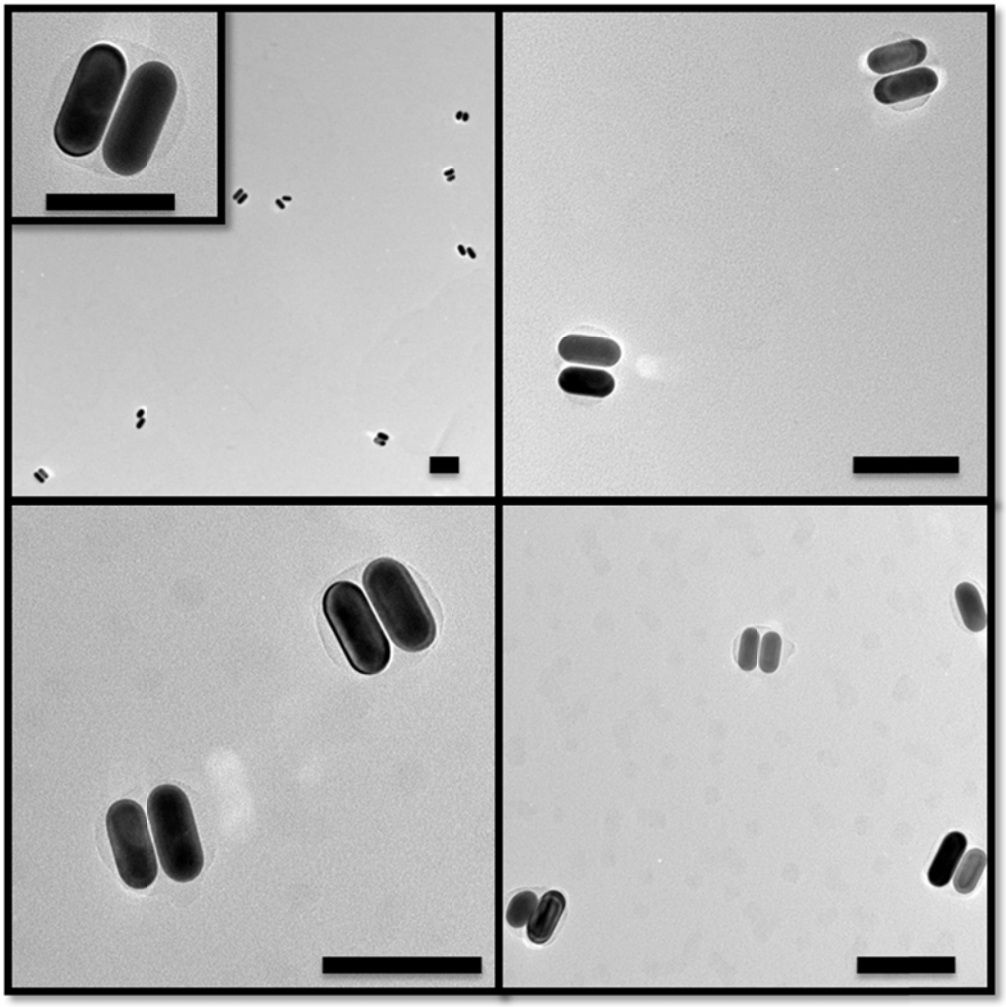

Supplementary Figure 25. TEM images of NR dimers with PS-PAA shells. Scale bars are 100 nm.

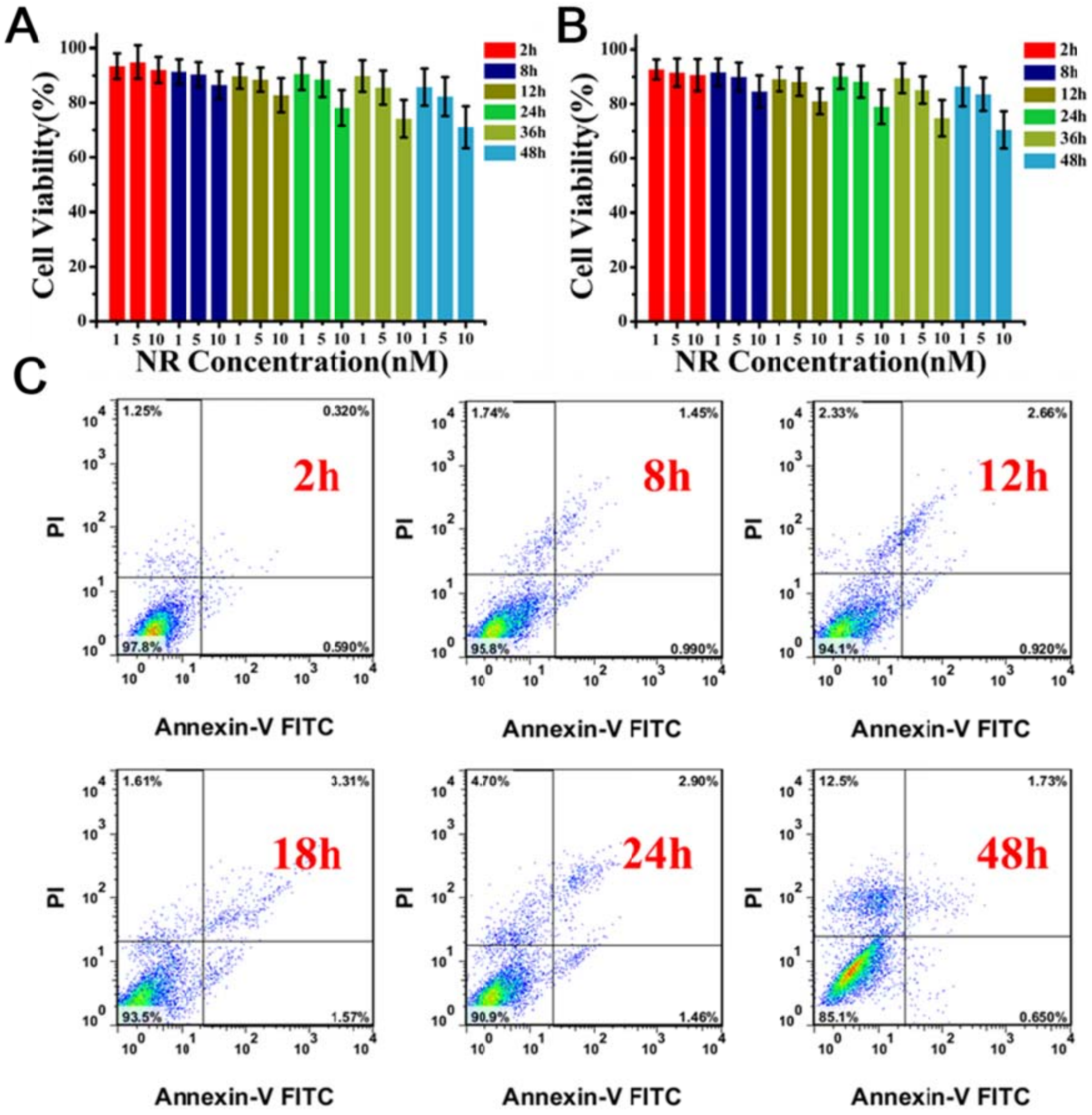

Supplementary Figure 26. Cell viability was measured by MTT kit (**A**) NR @PS-PAA, (**B**) NR dimers with PS-PAA shells, and (**C**) HeLa cells incubated with NR dimers with PS-PAA shells for various culture times, analyzed by flow cytometry. The error bars correspond to the standard error of the mean (n = 3).

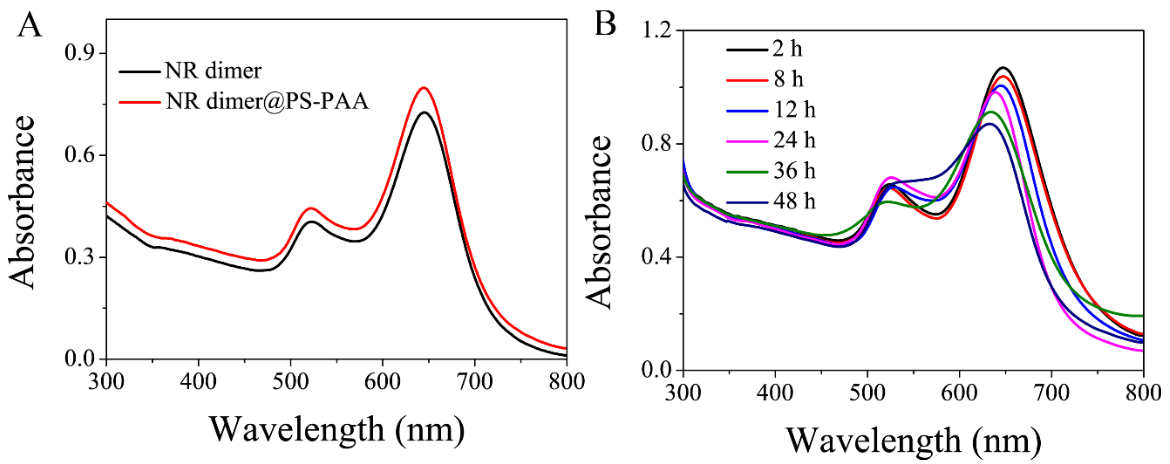

Supplementary Figure 27. UV-vis spectra of NR dimers with and without (**A**) PS-PAA shells in buffer and (**B**) in cell cultures of HeLa cells with incubation times ranging from 2 h to 48 h.

Comment: PS-PAA-coated dimers from NRs appear to be 'stiffer' than those from NPs. We attribute it to stronger vdW interactions and thicker polymer coating; formation of double bridges cannot be excluded.

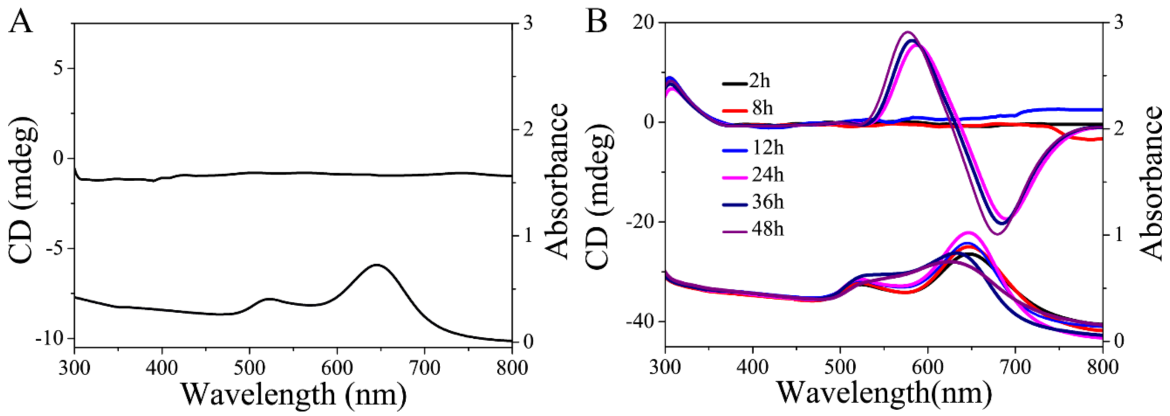

Supplementary Figure 28. CD (top) and UV (bottom) spectra of NRs with PS-PAA shells in (**A**) TBE buffer and (**B**) in HeLa cell cultures for different incubation times from 2 to 48 h. The emergence of the chiroptical activity after 24 h originates from the formation of NR assemblies inside the cells caused by agglomeration in vacuoles (exosomes) as in **Supplementary Figure 23**.

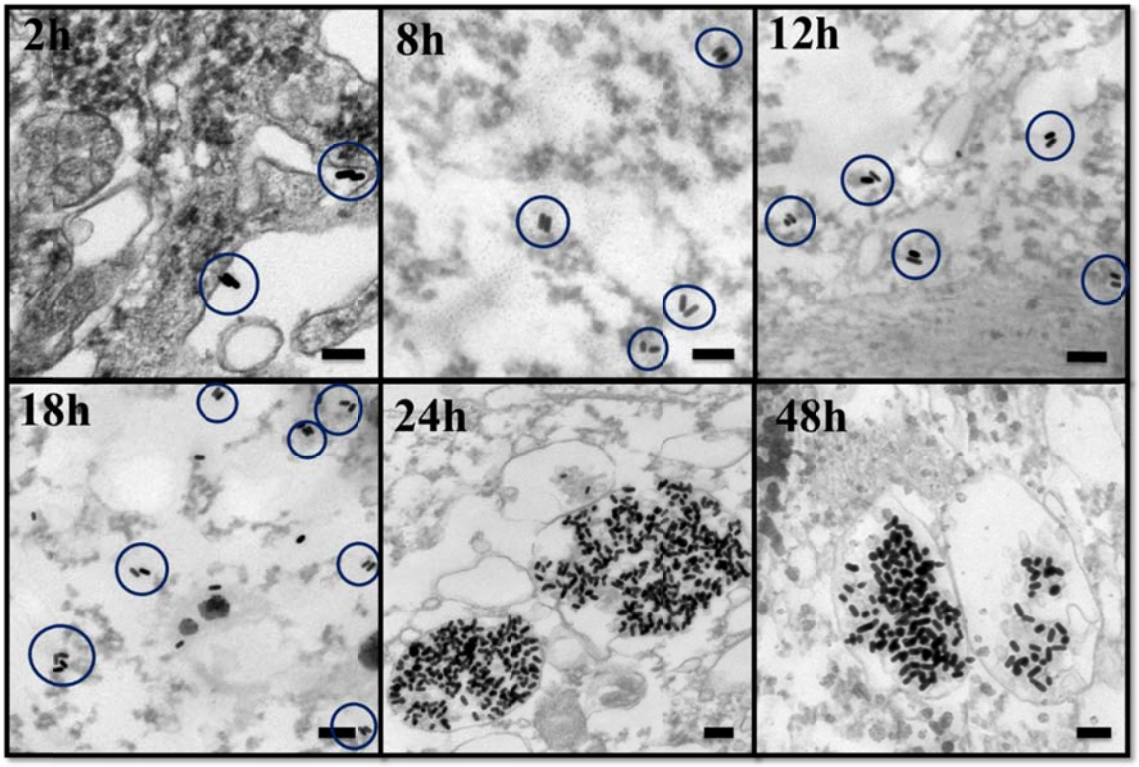

Supplementary Figure 29. Bio-TEM images of NR dimers with PS-PAA shells in HeLa cells for different incubation times from 2 h to 48 h indicated in the images; Scale bars are 200nm.

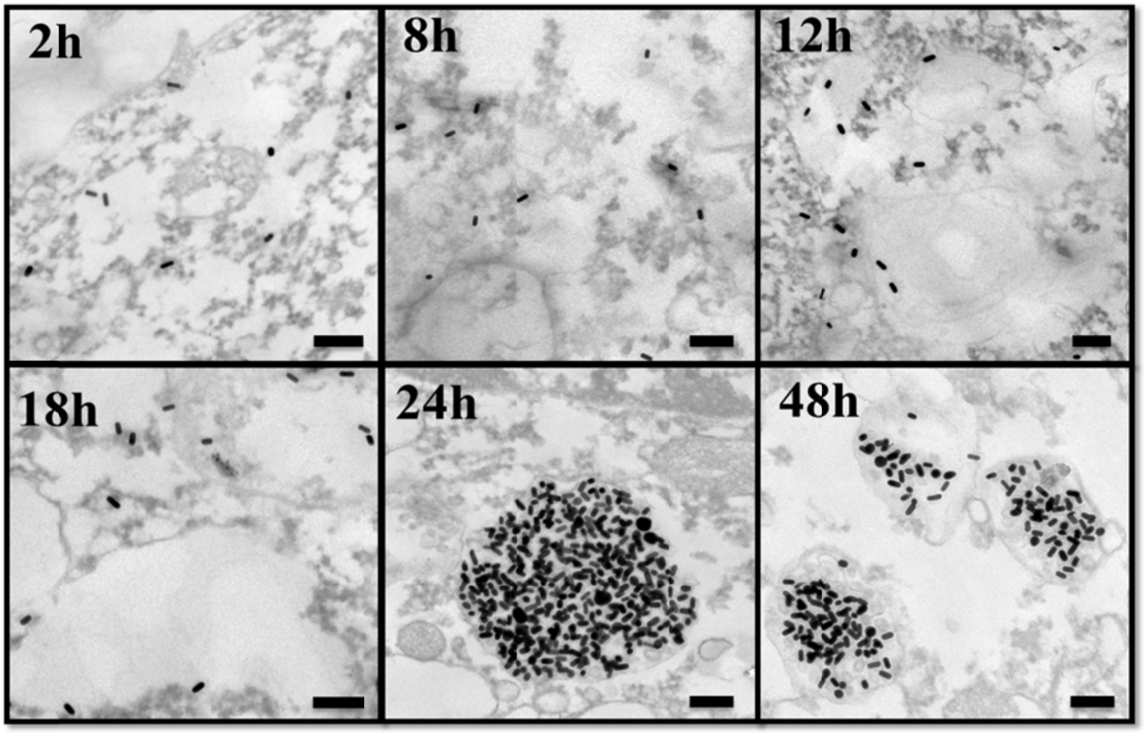

Supplementary Figure 30. Bio-TEM images of individual NRs with PS-PAA shells in HeLa cells with different incubation times from 2 h to 48 h indicated in the images; Scale bars are 200nm. The aggregation of individual NRs in the clusters after 24 h correlates with emergence of chiroptical activity in the 550-750 nm range as in **Supplementary Figure 28.**

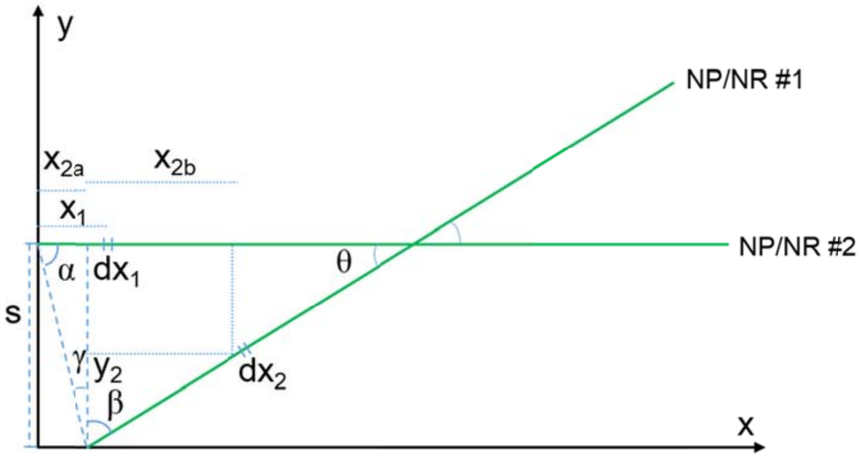

Supplementary Figure 31. The x-y plane view of the line charge representation of a twisted dimer from elongated NPs or NRs with dihedral angle θ. The additional *z*-axis for Supplementary Eq. 9 is orthogonal to this *x*-*y* plane and is parallel to the line connecting the centers of the NRs.

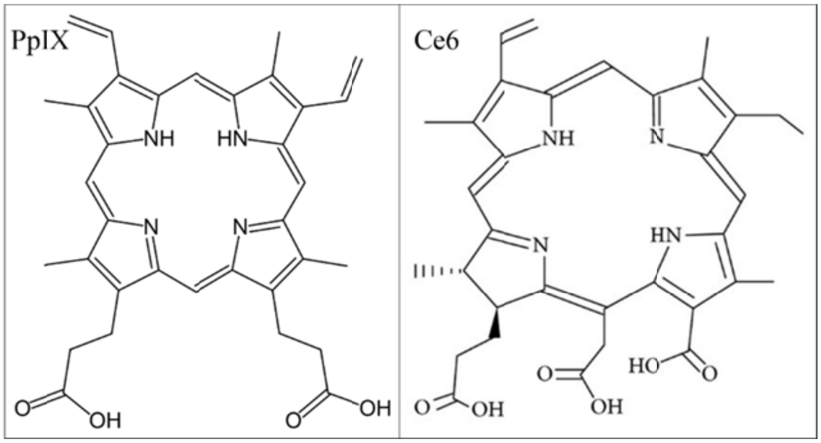

Supplementary Figure 32. Molecular structure of PpIX and Ce6 photosensitizers used in photodynamic therapy and in the experimental series in **Figures 4,5** in the main text and **Supplementary Figures 33-37** in this file with Supplementary Information.

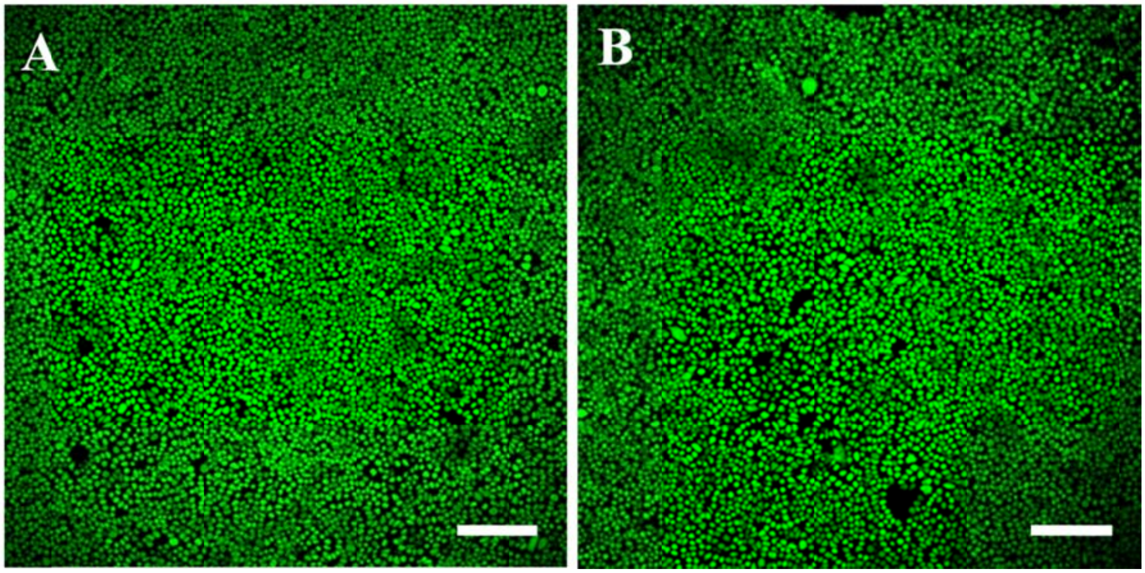

Supplementary Figure 33. Confocal fluorescence images of HeLa cells after (**A**) LCP, and (**B**) RCP irradiation at 532 nm, 5 mW/cm^2; 30 min illumination time. Scale bar 200 μm.

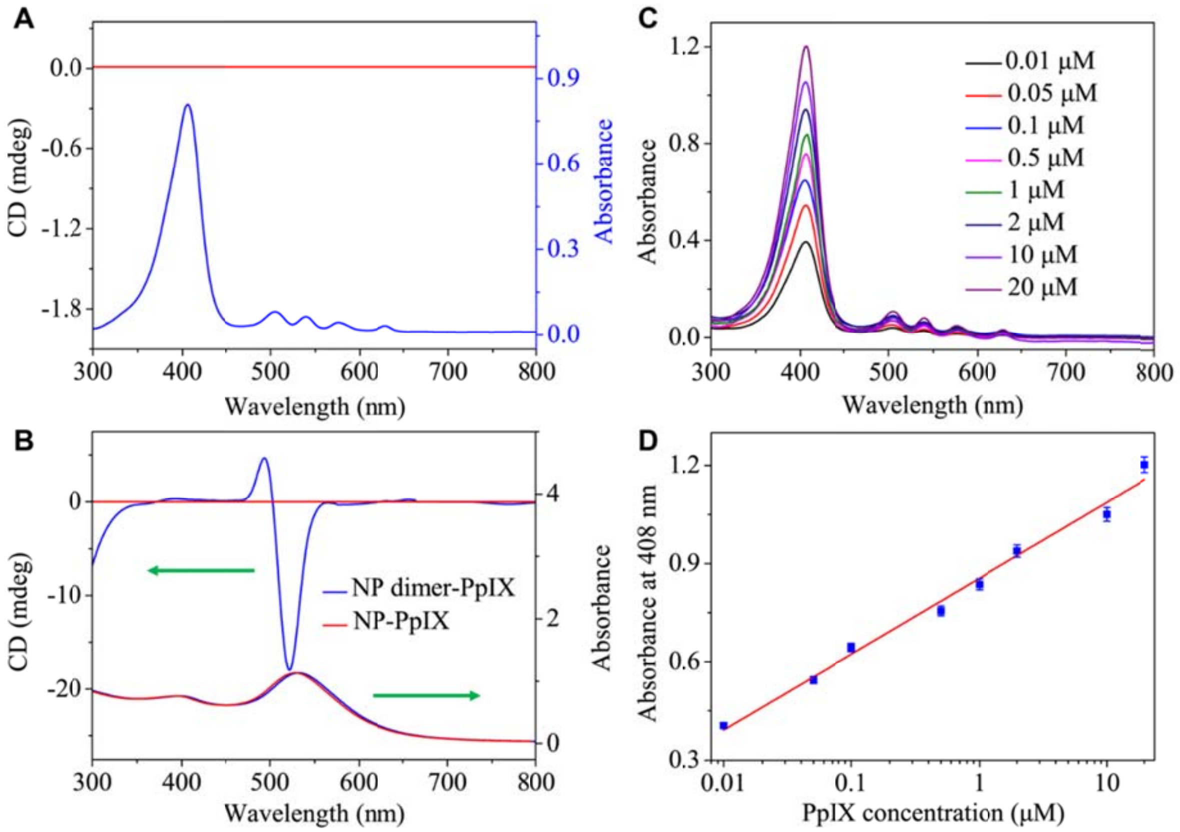

Supplementary Figure 34. CD (top) and UV-vis spectra (bottom) of (**A**) free PpIX, and (**B**) NPs and NP dimers after conjugation with PpIX. (**C**) UV-vis spectra of PpIX of different concentrations. (**D**) Calibration curve for concentration and absorbance of PpIX at 408 nm. The error bars correspond to the standard error of the mean (n = 3).

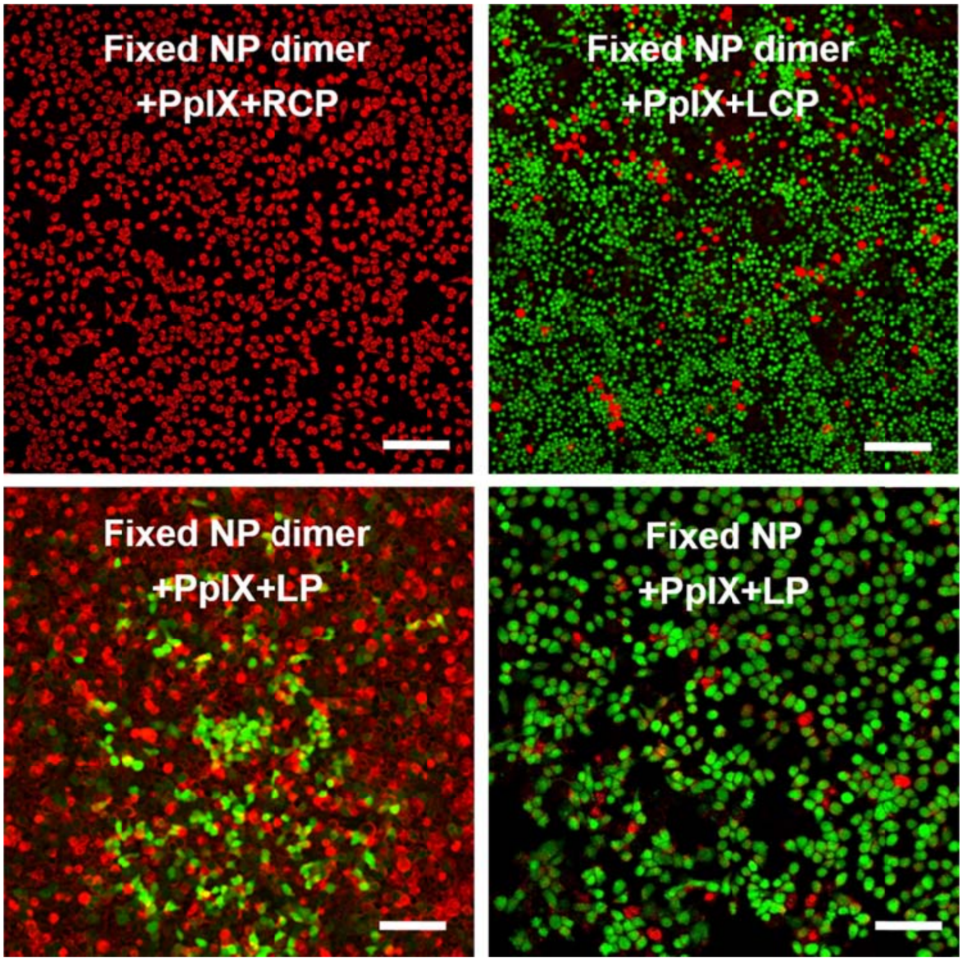

Supplementary Figure 35. The confocal images of HeLa cells incubated with 'fixed' NP dimer coated with PS-PAA after illumination by 532nm laser under different polarization conditions. Scale bar: 200 μm.

Comments: Cell viability was evaluated after coupling the PDT agent onto the nanoparticles. PpIX was conjugated to the NP dimers, while the Ce6 was used for NR dimers. Confocal images of NP dimer with PpIX and PS-PAA shell indicate that most of cells incubated with NP dimer with PS-PAA shell are killed under RCP illumination. While under LCP illumination, only a small part of cell is killed. The situation is opposite for NP dimer without PS-PAA shell, which demonstrated the configuration of NP dimer

with PS-PAA shell does not switch.

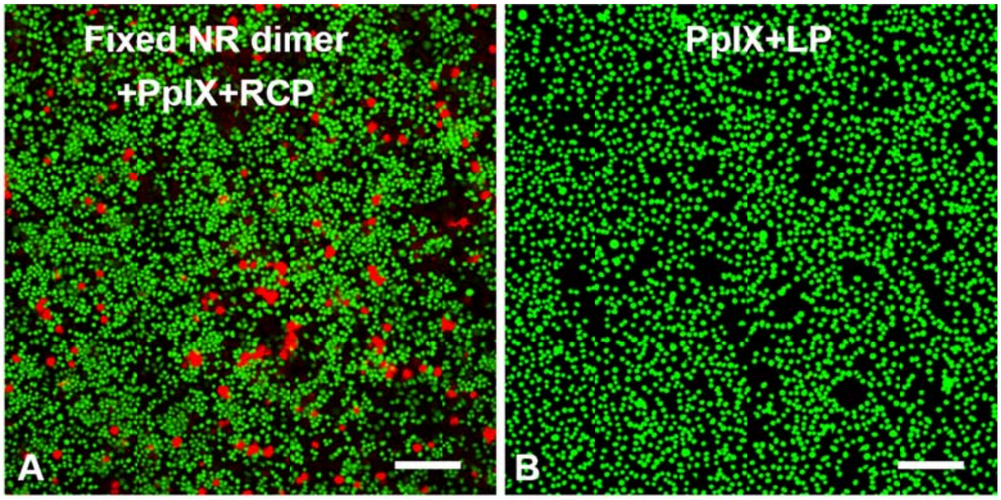

Supplementary Figure 36. The fluorescence microscopy images for the live/dead assays for HeLa cells after 30 min irradiation under (A) right-handed circular polarized (RCP) light for PS-PAA-constrained NR dimers conjugated with PpIX and (B) linearly polarized light (LP) for free PpIX with 660 nm photons (5 mW/cm^2). Scale bar: 200 μm.

Comment: The photodynamic therapy rate, that is photodynamic cell killing rate, was evaluated for PS-PAA-constrained (fixed) NR dimers conjugated with PpIX with 660 nm laser illumination as a control experiment. Similarly to **Supplementary Figure 34**, the cell incubated without NR dimers revealed >98% alive cells after irradiation with 660 nm laser, 5 mW/cm^2. A small amount of cell death could be observed in cell culture treated with PS-PAA-constrained NR dimers after 660 nm RCP (**Figure 5**). This cellular death was the much smaller than for dimers made from NPs and PpIX (**Figure 4A, Supplementary Figure 36** top left) because NRs and PpIX have almost no spectral overlap for 660 nm photons because the absorption band of PpIX at 630 nm is weak.

Note that circular polarization for incident light was chosen to maximize the adsorption inside the cells.

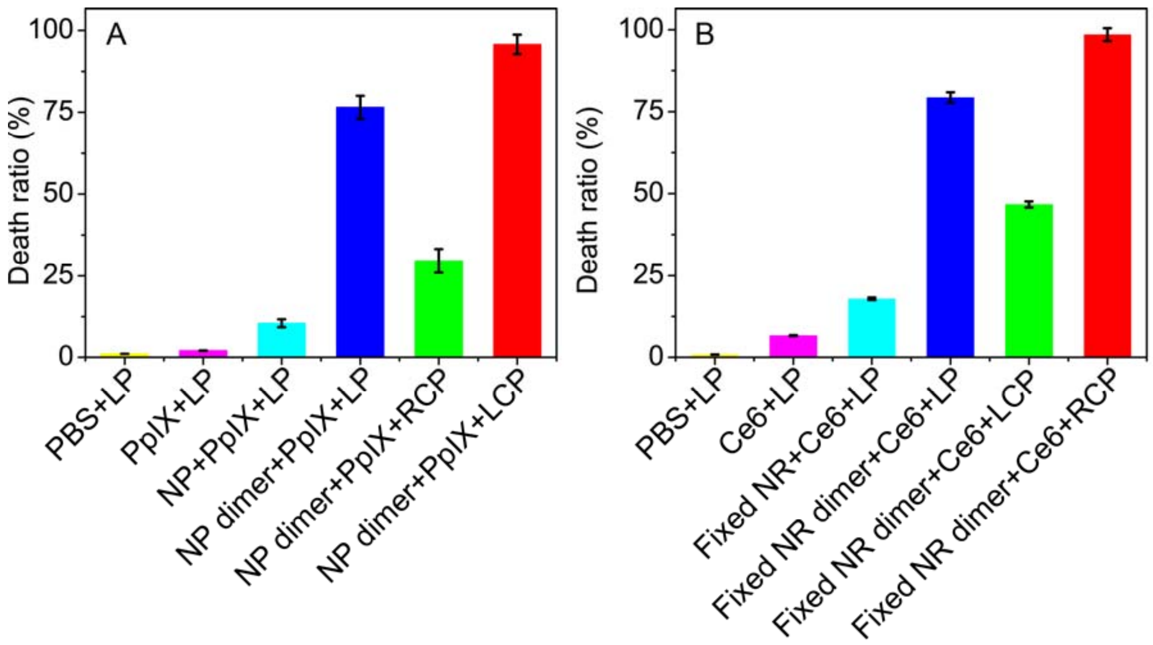

Supplementary Figure 37. The death ratio of HeLa cells incubated with (A) NP dimers, (B) NR dimers after irradiation under different polarization conditions. The error bars correspond to the standard error of the mean (n = 3).

Comment: The maximum cell death ratio for unconstrained NP pairs and constrained NR pairs is 93.5±3.5% and 94.5±2.5% under 532 nm LCP light and 660 nm RCP light, respectively. Furthermore, LCP light gives a three-fold higher death ratio in cancer cells compared with RCP light or LP light for unconstrained NP pairs, and the similar experiment for constrained NR could show that RCP light gives a higher percentage of apoptotic cells than LCP due to its stronger absorption by NR dimers and intracellular

localization of the dimers.

Supplementary References:

1. Frens, G. Controlled Nucleation for the Regulation of the Particle Size in Monodisperse Gold Suspensions. *Nat. Phys. Sci.* **241,** 20–22 (1973).

2. Xu, L. *et al.* New Synthesis Strategy for DNA Functional Gold Nanoparticles. *J. Phys. Chem. C* **115,** 3243–3249 (2011).

3. Wang, Y. *et al.* A systems approach towards the stoichiometry-controlled hetero-assembly of nanoparticles. *Nat. Commun.* **1,** 87 (2010).

4. Park, S. Y. *et al.* DNA-programmable nanoparticle crystallization. *Nature* **451,** 553–556 (2008).

5. Gore, J. *et al.* DNA overwinds when stretched. *Nature* **442,** 836–839 (2006).

6. Lee, O. S., Cho, V. Y. & Schatz, G. C. A- to B-form transition in DNA between gold surfaces. *J. Phys. Chem. B* **116,** 7000–7005 (2012).

7. Makino, K. & Ohshima, H. Electrophoretic mobility of a colloidal particle with constant surface charge density. *Langmuir* **26,** 18016–18019 (2010).

8. Sadelain, M., Papapetrou, E. P. & Bushman, F. D. Safe harbours for the integration of new DNA in the human genome. *Nat Rev Cancer* **12,** 51–58 (2012).

9. Shen, X. *et al.* 3D plasmonic chiral colloids. *Nanoscale* **6,** 2077–2081 (2014).

10. Besteiro, L. V. & Govorov, A. O. Amplified Generation of Hot Electrons and Quantum Surface Effects in Nanoparticle Dimers with Plasmonic Hot Spots. *J. Phys. Chem. C* **120,** 19329–19339 (2016).

11. Cecconello, A. *et al.* DNA Scaffolds for the Dictated Assembly of Left-/Right-Handed Plasmonic Au NP Helices with Programmed Chiro-Optical Properties. *J. Am. Chem. Soc.* **138,** 9895–9901 (2016).

12. Levi-Belenkova, T., Govorov, A. O. & Markovich, G. Orientation-Sensitive Peptide-Induced Plasmonic Circular Dichroism in Silver Nanocubes. *J. Phys. Chem. C* **120,** 12751–12756 (2016).

13. Kuzyk, A. *et al.* A light-driven three-dimensional plasmonic nanosystem that translates molecular motion into reversible chiroptical function. *Nat. Commun.* **7,** 10591 (2016).

14. Elstner, M., Hobza, P., Frauenheim, T., Suhai, S. & Kaxiras, E. Hydrogen bonding and stacking interactions of nucleic acid base pairs: A density-functional-theory based treatment. *J. Chem. Phys.* **114,** 5149–5155 (2001).

15. Zhang, H. & Govorov, a. O. Giant circular dichroism of a molecule in a region of strong plasmon resonances between two neighboring gold nanocrystals. *Phys. Rev. B* **87,** 075410 (2013).

16. Pino, P. del *et al.* Protein corona formation around nanoparticles – from the past to the future. *Mater. Horiz.* **1,** 301–313 (2014).

17. Lundqvist, M. *et al.* Nanoparticle size and surface properties determine the protein corona with possible implications for biological impacts. *Proc. Natl. Acad. Sci. U. S. A.* **105,** 14265–14270 (2008).

18. Cifuentes-Rius, A., Puig, H. de, Kah, J. C. Y., Borros, S. & Hamad-Schifferli, K. Optimizing the Properties of the Protein Corona Surrounding Nanoparticles for Tuning Payload Release. *ACS Nano* **7,** 10066–10074 (2013).

19. Camden, J. P. *et al.* Probing the Structure of Single-Molecule Surface-Enhanced Raman Scattering Hot Spots. *J. Am. Chem. Soc.* **130,** 12616–12617 (2008).

20. Fang, Y., Seong, N.-H. & Dlott, D. D. Measurement of the Distribution of Site Enhancements in Surface-Enhanced Raman Scattering. *Science (80-.).* **321,** 388-392 (2008).

21. Samoc, M., Samoc, A. & Grote, J. G. Complex nonlinear refractive index of DNA. *Chem. Phys. Lett.* **431,** 132–134 (2006).

22. Cohen, G. & Eisenberg, H. Deoxyribonueleate solutions: Sedimentation in a density gradient, partial specific volumes, density and refractive index increments, and preferential interactions. *Biopolymers* **6,** 1077–1100 (1968).

23. Banihashemian, S., Periasamy, V., Mohammadi, S., Ritikos, R. & Rahman, S. Optical Characterization of Oligonucleotide DNA Influenced by Magnetic Fields. *Molecules* **18,** 11797–11808 (2013).
